# Translational Arrest Due to Cytoplasmic Redox Stress Delays Adaptation to Growth on Methanol and Heterologous Protein Expression in a Typical Fed-Batch Culture of *Pichia pastoris*

**DOI:** 10.1371/journal.pone.0119637

**Published:** 2015-03-18

**Authors:** Bryn Edwards-Jones, Rochelle Aw, Geraint R. Barton, Gregory D. Tredwell, Jacob G. Bundy, David J. Leak

**Affiliations:** 1 Department of Life Sciences, Faculty of Natural Sciences, Imperial College London, London, SW7 2AZ, United Kingdom; 2 Department of Surgery & Cancer, Faculty of Medicine, Imperial College London, London, SW7 2AZ, United Kingdom; 3 Department of Biology & Biochemistry, University of Bath, Claverton Down, Bath BA2 7AY, United Kingdom; University of Delhi, INDIA

## Abstract

**Results:**

We have followed a typical fed-batch induction regime for heterologous protein production under the control of the *AOX1* promoter using both microarray and metabolomic analysis. The genetic constructs involved 1 and 3 copies of the *TRY1* gene, encoding human trypsinogen. In small-scale laboratory cultures, expression of the 3 copy-number construct induced the unfolded protein response (UPR) sufficiently that titres of extracellular trypsinogen were lower in the 3-copy construct than with the 1-copy construct. In the fed-batch-culture, a similar pattern was observed, with higher expression from the 1-copy construct, but in this case there was no significant induction of UPR with the 3-copy strain. Analysis of the microarray and metabolomic information indicates that the 3-copy strain was undergoing cytoplasmic redox stress at the point of induction with methanol. In this Crabtree-negative yeast, this redox stress appeared to delay the adaptation to growth on methanol and supressed heterologous protein production, probably due to a block in translation.

**Conclusion:**

Although redox imbalance as a result of artificially imposed hypoxia has previously been described, this is the first time that it has been characterised as a result of a transient metabolic imbalance and shown to involve a stress response which can lead to translational arrest. Without detailed analysis of the underlying processes it could easily have been mis-interpreted as secretion stress, transmitted through the UPR.

## Introduction

The methylotrophic yeast, *Pichia pastoris* is a commonly used host for protein expression [1, 2]. High volumetric productivities of both secreted and intracellular proteins can be produced, largely because of the high cell densities achievable in fed-batch fermentation. Specific productivities, particularly of secreted proteins, are, in fact, relatively modest [3] because of the limited flux through the eukaryotic protein export system. However, as growth and induction is typically done in a minimal medium and the host strain naturally secretes very little protein, this protein is usually fairly pure.

Expression typically uses the very strong methanol-inducible alcohol oxidase promoter *AOX1* which, in the parental strain, is capable of inducing alcohol oxidase production up to 30% of the total intracellular protein [1, 4]. Genes to be expressed are chromosomally integrated downstream of *AOX1* by either recombination at a single site, generating a mut^+^ (methanol utilisation positive) strain, in which a functional copy of the *AOX1* gene is retained, or transplacement, in which the *AOX1* gene is disrupted. This can yield a mut^s^ (slow) or mut^-^ strain depending on the availability of a functional *AOX2* gene which encodes a second alcohol oxidase with allows slow growth on methanol [5]. Production is usually done in fed-batch in which cells are grown to high densities using glycerol as the carbon source, induced initially with a pulse of methanol and then switched to a continuous methanol feed which may be maintained for up to 96h [1].

Despite the impressive track record of this expression system and occasional high titres recorded, the levels of expression are relatively poor compared to the evident strength of the *AOX1* promoter [1], and lengthy induction periods are needed to give good protein yields. With secreted proteins, which form the bulk of the examples in the literature, at least part of the problem has been ascribed to the effects of exceeding the secretory capacity of the cell [6, 7]. The folding of secreted proteins in the ER is assisted by chaperones such as Kar2p and Pdi. High demand for Kar2p caused either by relatively high rates of transit or poor folding, as is frequently found with heterologous proteins, induces the unfolded protein response (UPR), which increases chaperone production [8]. However, exceeding the capacity of the UPR to beneficially affect secretion leads to increased ER associated degradation (ERAD) of the heterologous protein, which can actually reduce secretion to levels below those achieved at moderate levels of expression [6].

One problem with the *AOX1* induction system is that, particularly in mut+ strains, induction coincides with a major reorganisation of metabolism, not only requiring novel catabolic functions for assimilation of formaldehyde, but a massive proliferation of peroxisomes [9] which are the location of alcohol oxidase and catalase (which breaks down the H_2_O_2_ generated by alcohol oxidase). Until the methanol metabolic machinery is in place and even beyond this point if cell proliferation continues, the production of peroxisomes in particular is likely to act as a drain on cellular resources. Recently, a few studies have compared the transcriptomes of recombinant (expressing) and non-recombinant *P*. *pastoris* cells sampled either at single time points after induction of expression [10] or grown in chemostat culture [11, 12]. While the comparison is informative, the use of chemostat culture (primarily for better reproducibility of data) does not capture the dynamics of a typical industrial production process. Therefore, although the data obtained is inherently more noisy, in order to explore the events occurring during the early stages of induction both in wild type and recombinant strains we have followed a typical fed-batch induction regime using transcriptomic and metabolomic analyses. This was done using human trypsinogen as the recombinant protein as this has previously been characterised as producing copy-number dependent UPR and enhanced ERAD [6]. This facilitated both a progressive time course analysis for each strain and side by side comparisons between wild-type and recombinant strains at the same times post-induction.

## Materials and Methods

### Strain construction

*P*. *pastoris* GS115 (*HIS4*-91), recently reclassified as *Komagataella phaffii* [13], was obtained from Invitrogen, Paisley, UK. A 5’ truncated gene encoding 722bp of the human trypsinogen-1 gene (*TRY1*) starting at base 63 of the coding region after the signal peptide sequence and flanked by SfiI sites, was synthesized by Genscript (Piscataway, NJ, USA) and supplied in pUC57. *TRY1* was released from pUC57 with SfiI, gel purified and inserted into the SfiI site of pPICz α B (Invitrogen) placing the gene in frame with the α-factor signal peptide in the vector. A stop codon had been inserted into the synthetic gene so that translation did not read through into the vector *HIS* and *MYC* tag sequences. Recombinants were initially selected in *E coli* JM109 grown on LB containing 100μg ml^−1^ Zeocin and authenticity confirmed by sequencing. For cloning into *P*. *pastoris*, recombinant plasmid was isolated from *E coli*, linearised by digestion with PmeI, which cuts in the 5’ *AOX1* promoter region and electroporated into *P*. *pastoris* GS115. Recombinant colonies were selected after 4 days incubation on YPD containing 100μg ml^−1^ Zeocin, grown on YPD plates without Zeocin then on YPD containing Zeocin, to ensure that Zeocin resistance was not transient. The presence of a single *TRY1* vector insert in selected clones was confirmed by Southern blotting of BamHI digested DNA separated on a 1% agarose gel, with a DIG labelled *TRY1* probe.

Two and three *TRY1* gene copy recombinant strains were made using the BglII and BamHI sites which flanked the pPICz α B*TRY1* expression cassette (promoter, alpha factor signal peptide, *TRY1* and 3' UTR region). Thus, pPICz α B*TRY1* was digested with BglII and BamHI and the 2.5kb expression cassette fragment gel purified and ligated together in the absence of vector to form multimers. The ligation products were cut with BglII and BamHI to remove any “head to head” or “tail to tail” fragments and products of sizes consistent with containing 2 and 3 copies of the *TRY1* cassette were separated on a 1% agarose gel and purified. Individual purified products were then ligated into BglII and BamHI digested pPICz α B, before transformation into *E coli*. Confirmation of vector construction was done by restriction digestion with BglII and BamHI to release the vector backbone and *TRY1* expression cassettes. The 2 and 3 copy TYRY1 expression vectors were introduced into *P*. *pastoris* GS115 as described above and confirmed by Southern blotting with a DIG labelled *TRY1* probe.

### Expression in tubes

Single colonies were inoculated into 5 mL of BMGY (1% (w/v) Yeast Extract, 2% (w/v) Peptone, 100mM potassium phosphate, pH6.0, 1.34% (w/v) Yeast Nitrogen Base (YNB), 4 x 10^–5^% (w/v) d-Biotin, 1% (v/v) glycerol) in 50ml centrifuge tubes and grown at 30°C, 250 rpm for 24 hours, with the tube lid loosely attached for gas exchange. The OD600 was measured and cultures normalised by taking a volume of cells equivalent to that contained in 5 ml of OD600 10. Cells were centrifuged for 5 minutes at 4000 rpm at room temperature and the supernatant removed. The cultures were then resuspended in methanol containing BMMY (as BMGY but with 0.5% (v/v) methanol replacing glycerol) and left to grow for a further 24 hours.

### Fed-batch culture

Fed-batch culture was performed in a 7 L fermentor (Applikon, Netherlands) at an initial batch working volume of 4 L. Only the wild type (GS115), *TRY1*-1 copy and *TRY1*-3 copy were grown in fed-batch culture and each strain was grown in duplicate. The initial glycerol batch phase was performed in basal salts medium, which was inoculated from a 30 ml overnight YPD culture. Upon depletion of the glycerol a 50% v/v glycerol feed was initiated at 0.06 L/hr until the culture reached OD_600_ 80 which triggered the end of the glycerol feed and a 0.5% v/v bolus addition of methanol. The methanol feed was controlled by a Raven methanol probe (Raven Biotech Inc, Canada) which maintained the methanol concentration at 0.5% and was maintained for 24hrs after the bolus addition.

### Media

Basal salt medium contained per litre: 9 ml phosphoric acid (85%), 0.2 g CaSO_4_, 6 g K_2_SO_4_, 4.7 g MgSO_4_.7H_2_O, 7.5 g KOH, 5 g (NH_4_)_2_SO_4_, 20 ml glycerol, 1 ml Acepol-83E (10% solution), 3 ml PTM4, 20 ml 2% w/v histidine. The basal media was heat sterilised *in situ* and the PTM4 and histidine added as a post sterile addition. PTM4 Trace Elements Solution contained per litre 2 g CuSO_4_.5H_2_O, 0.08 g NaI, 3 g MnSO_4_.4H_2_O, 0.2 g Na_2_MoO_4_.2H_2_O, 0.02 g boric acid, 0.5 g CaSO_4_.2H_2_O, 0.5 g CoCl_2_, 7 g ZnCl_2_, 22 g FeSO_4_.7H_2_O, 0.2 g biotin, and 1ml concentrated sulphuric acid. On starting the glycerol feed the medium was supplemented with (per L) 2.5 ml 2% w/v histidine and 1 ml PTM4. The methanol feed bottle contained 400ml methanol and 4.8ml of PTM4. pH was controlled at pH5 with 28% (w/v) ammonium hydroxide, stirrer speed at 1000rpm and dissolved oxygen tension at 35% using air mass flow control from 1.3L/min to 8L/min.

### Sampling

The fed-batch culture was sampled at 0h (pre-induction), 0.5h, 1h, 2h, 3h, 4h, 5h, 7h and 24h post-introduction of the methanol feed. All samples were used for metabolomics analysis, but only samples taken at 0h, 2h, 4h and (*TRY1*-1 strains only) 24h were used for microarray analysis. Time zero sample was taken before the glycerol feed was stopped and immediately before the addition of methanol.

For transcriptome analysis 1ml of culture was added to 9 ml RNA-later (Ambion) in order to stabilise the transcripts. This mixture was then centrifuged at 4000 rpm (Eppendorf 5810R) for 5 mins and the pellets re-suspended in 1 ml RNA-later and stored at −80°C until required.

Sampling from the bioreactor for metabolomics samples was performed as previously described, using a total culture extraction [14]. Briefly, a cell suspension (∼2 ml) from the bioreactor was sampled rapidly (under reduced pressure) into a cold (<−50°C) aqueous methanol (60% v/v final conc., 13 ml). The solutions were mixed thoroughly and frozen in liquid nitrogen. The sample was then thawed in an ultrasonic bath for 15 min and centrifuged for 5 min at 5000 g. The extract supernatant was concentrated under reduced pressure and samples were stored at −80°C until analysis. At the same sampling time 1 ml of the cell suspension from the bioreactor was sampled into a 1.5ml centrifuge tube to collect the extracellular metabolites. The sample was immediately centrifuged for 5 min at 16000 g and the supernatants were transferred to a new tube, concentrated under reduced pressure and stored at −80°C until analysis.

### NMR analysis

Samples were resuspended in 0.6 ml NMR buffer consisting of 0.1 M phosphate buffer pH 7.4, 1 mM trimethylsilyl-2,2,3,3-tetradeuteropropionic acid (TSP), and 0.55 ml was transferred to a 5 mm NMR tube. Spectra were acquired on a Bruker Avance DRX600 NMR spectrometer (Bruker BioSpin, Rheinstetten, Germany), with 1H frequency of 600 MHz, and a 5 mm inverse probe. Samples were introduced with an automatic sampler and spectra were acquired following the procedure described by Beckonert *et al* [15]. Briefly, a one- dimensional NOESY sequence was used for water suppression; data were acquired into 64 K data points over a spectral width of 12 KHz, with 8 dummy scans and 512 scans per sample. In addition, 2D ^1^H-^1^H COSY were acquired. Data were acquired into 512 × 4096 data points covering 6 × 6 KHz, with 3 scans for each increment.

Spectra were processed in iNMR 2.6.3 (Nucleomatica, Molfetta, Italy). Fourier transform of the free-induction decay was applied with a line broadening of 0.5 Hz. Spectra were manually phased and automated first order baseline correction was applied. Metabolite assignments were based on Tredwell et al [14]. Metabolite concentrations from 2D spectra, relative to the internal standard TSP were calculated using rNMR [16], and data were normalised by the probabilistic quotient normalisation method described by Dieterle et al [17].

### Array design

An Agilent (Agilent, Santa Clara) 4 × 44,000 probe custom microarray was designed by Oxford Genome Technologies (OGT, Oxford, UK) based on the Integrated Genomics *P*. *pastoris* GS115 genome, which contained 5195 open reading frames (ORFs). Where possible, 9 non-overlapping probes per ORF were designed, taking into account uniqueness, GC content, predicted hybridisation conditions and minimizing G-spots [18], see Table 1 for summary of the number of probes per gene. In the cases where only a single unique probe could be designed, further overlapping probes were designed. 5168 out of the 5195 ORFs had at least one unique probe mapping to it. It was not possible to design at least one unique probe for 27 of the ORfs. Blasting all probes against the genome revealed 384 of the designed probes had the potential to cross hybridise. These probes have been highlighted with an ‘ortho’ appended to the identifier. The array includes positive spike-in control probes and negative probes. In total 39,412 probes were designed.

**Table 1:** The number of unique probes on the micro-array per gene.

Number of Genes	Number of Probes	Avg. Gene Length
78	1	158
183	2	224
197	3	329
210	4	430
281	5	519
275	6	615
275	7	712
298	8	808
3371	9	1844

A total of 3371 genes were targeted by 9 unique probes, with the average length of these genes being 1844 bases.

Subsequent to the design of the array presented here, De Schutter *et al* [19] published a version of the *P*. *pastoris* genome. A comparative analysis was performed to determine how well the probes from this array matched the De Schutter genome using Blast. Out of the 39,412 probes, 38,552 (97.8%) mapped to one position, 845 (2.1%) did not map anywhere, and 15 (0.04%) mapped at two or more loci.

### Sample processing for microarray analysis

RNA was prepared using RiboPure Yeast RNA kit (Ambion) according to the manufacturer’s instructions using 200μl of the stored sample. The samples were treated with the supplied DNAse and the absence of genomic DNA was confirmed by qPCR using primers to *ACT1* and the 2X SYBR Green JumpStart Taq Ready Mix (Qiagen, Crawley, UK) qPCR kit. RNA quantity and quality was assessed using the Bio-Rad Experion system and the Stdsens kit. RNA of >200 μg/ml, RQI of >7 and OD_*260/280*_ ratio >1.8 and OD_*260/230*_ ratio > 1.5 were submitted for transcriptomic analysis.

### Analysis of microarray data

Microarray data are available in the ArrayExpress database (www.ebi.ac.uk/arrayexpress) under accession number E-MTAB-2530. Analysis was carried out in the R programming language using Bioconductor package to identify differentially expressed genes. Each of the time point samples for a particular strain was contrasted to the time zero sample for that strain and, additionally, samples of the *TRY1*-3 and *TRY1*-1 strains taken at a fixed time point were contrasted to each other and the non-recombinant GS115 in order to reveal any statistically significant differences. The Empirical Bayes method was applied to identify statistical significance in contrast between gene expression profiles [20]. The false discovery rate (fdr) based on Benjamini and Hochberg’s method, which assumes that all genes are statistically different from one another was set to be less than 5% [21]. Gene functionality was assigned with reference to a curated *P*. *pastoris* genome which can be viewed in a gBrowse [22] instance hosted by the Bioinformatics Support Service, Imperial College London (www.blugen.org/gbrowse-bin/gbrowse/Pichia/).

### Heat Maps and Pathway Analysis

Heat maps of log 2 normalised expression levels were generated using the Heatplus.2 function from the R-bioconductor package. Genes with similar expression levels were clustered and presented as trees, using the same package. Pathway analysis was also used to identify sequential metabolic processes, which were significantly up or down-regulated in accordance with the gene expression data. Initially significantly up- or down-regulated genes were run through KOBAS (KEGG Orthology Based Annotation System), which assigned genes to pathways based on the KEGG maps specifically for *P*. *pastoris* [23–25]. Once pathways had been identified the KEGG Search & Colour pathway was used to visually map the differentially expressed genes [26, 27]. Given the inherent noise in the system, discussion of regulation focuses on those pathways in which all/virtually all changes were in a consistent direction (up or down-regulation).

## Results

### Preliminary tests of genetic constructs

The effects of expression of multiple copies of *TRY1* have been previously described, but as these constructs were not made available to us we needed to confirm that our constructs performed in a similar manner. Recombinant strains (3 of each) containing 1, 2 and 3 copies of the *TRY1* expression cassette were grown in 50ml tube cultures and supernatants analysed for Try1p expression using an Experion Pro260 protein analysis chip (BioRad). This showed the expected pattern of increasing (but not doubled) expression going from 1 to 2 copy numbers, but lower expression (approx. 50%) in the 3 copy number strain than observed in the 1 copy number strain. The correlation between reduced expression and secretion stress leading to the induction of UPR was also demonstrated by qRT-PCR of *HAC1* expression [28] which was 3–4 fold higher in the 3 copy number strains than in 1 copy number strains) (S1).

### Growth characteristics in fed-batch culture

GS115 and recombinant strains carrying 1 (*TRY1*-1) and 3 (*TRY1*-3) copies of *TRY1* were grown in duplicate fed-batch cultures involving initial growth on glycerol to increase biomass, followed by a switch to methanol fed-batch growth, with methanol concentration measured with a dedicated probe and controlled at approximately 0.5% (v/v), throughout in order to avoid the metabolic stress that has been associated with high methanol concentrations [29]. Aeration was started at 1L/min and the dissolved oxygen concentration allowed to drop to 30% (saturation = 100%) where it was maintained by increasing the aeration rate. For GS115 this produced a biphasic growth profile with oxygen demand increasing rapidly after the addition of methanol to a maximum level between 3 and 7h after induction. The profile for *TRY1*-1 was characterised by a reduced rate of biomass increase in the second phase, after addition of methanol, while *TRY1*-3 exhibited virtually no biomass increase for at least 24h after addition of methanol, although methanol metabolism was clearly occurring.

Samples for both microarray and metabolomic analysis were taken as described in methods. The time zero sample was taken prior to the addition of methanol, when cells were growing in fed-batch on limiting amounts of glycerol. The experimental design allowed insight into four underlying processes: 1) the changes occurring in GS115 during the switch from glycerol to methanol; 2) comparison between *TRY1*-1 and GS115 during induction of heterologous protein expression; 3) the consequence of massive secretion stress on induction in *TRY1*-3, and 4) comparison of *TRY1*-1 and GS115 pre-induction. In this paper we focus on the significant major processes and differences between constructs and time points. Additional information is provided as supplementary data.

### Transcriptional and metabolomic profiles in methanol fed-batch culture

#### Characterisation of the underlying effect of growth rate changes

Even in the wild-type GS115 the switch from glycerol to methanol as the sole carbon substrate involves a reduction in growth rate, which was more marked in the *TRY1*-1 and *TRY1*-3 strains. In *S*. *cerevisiae* reduction of growth rate has been shown to induce transcriptional responses similar to those induced by nutritional starvation [30]. This includes reduction in protein synthesis capability and DNA replication, and an increase in autophagy. Ribosome production has been estimated to use approx. 90% of the total cell energy in exponentially growing yeast [31] so it is not surprising that this is rapidly down-regulated in response to a reduction in growth rate. Binding sites for RNA polymerases I (A) and III (C) are upstream of ribosome biogenesis (Ribi) genes and the down-regulation of expression of these polymerases could partly account for reduced Ribi gene expression [32].

#### DNA, RNA and protein synthesis

In all three strains there was a major reduction in expression of Ribi genes and ribosomal protein production within the first 2h after introduction of methanol, together with a reduction in RNA degradation (core exosome) activity. Consistent with the reduction of growth rate, genes encoding most of the DNA replication machinery were down-regulated and cell cycle related functions re-adjusted. While many of these effects returned to their basal level 4h after the start of methanol feeding, ribosomal biogenesis genes remained down-regulated at 4h, consistent with the lower growth rate on methanol compared to glycerol (S2 Fig.). This confirms that switching to growth on methanol is classically diauxic, causing a transient starvation while the methanol metabolic machinery is induced, followed by adaptation to a new, lower growth rate. Despite the increased metabolic load of heterologous protein production there was little difference in the down-regulation in ribosomal protein production (S3 Fig.) and ribosomal biogenesis (S2 Fig.) between *TRY1*-1 and GS115, when the two were directly compared at 2h post-methanol addition. However, consistent with the complete cessation of growth, ribosomal protein gene expression in *TRY1*-3 was lower than both *TRY1*-1 and GS115, 2h after methanol addition (S3 Fig.). The same pattern was also observed with nuclear pore transport and some translation initiation factors. This pattern of reduced expression in *TRY1*-3 was similar after 4h, with ribosomal biogenesis and ribosomal protein synthesis being down-regulated in comparison to *TRY1*-1.

**Fig 1:**
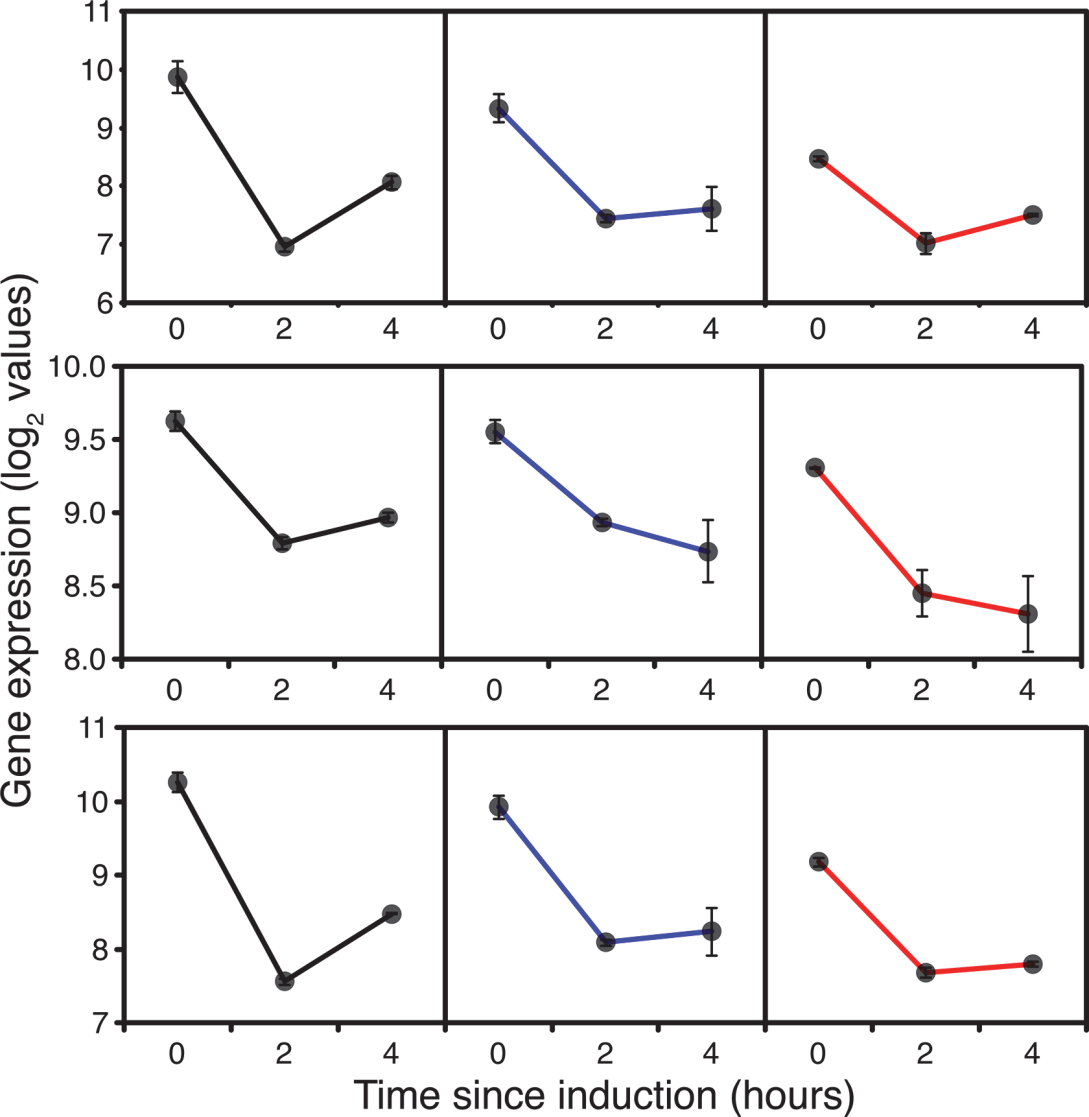
Expression of RNA polymerase I, II and III core function genes, 0 (before), 2 and 4 hours after the start of methanol addition to wild-type GS115 (black), *TRY1-1*(blue) and *TRY1-3* (red), strains containing 1 and 3 gene copies of the human typsinogen gene, respectively, under the control of the *AOX1* promoter. From top to bottom, panels represent *RPA1*, *RPB2* and *RPC2*. Error bars show SEM.

Surprisingly, in addition to the down-regulation of expression of RNA pol I and III subunits, some RNA pol II core and specific subunits were down-regulated in all strains 2h after switching to methanol (S4 Fig. PAS_chr2-1_0125, chr4_0906, chr3_0244, chr1-4_0359), indicating a reduction in net protein synthesis. This is not typically linked to growth rate [33] but significant in the context of heterologous protein expression. However, by 4h expression of all RNA polymerase genes was starting to pick up in GS115, consistent with adaptation to a lower growth rate on methanol in GS115. Compared to their levels at 2h of induction many of the tRNA synthases were also up-regulated at 4h supporting the indication from pol II dynamics for a greater demand for net protein synthesis. However, in *TRY1*-3 in addition to reduction in expression of RNA polymerase I and III subunits, some core and specific RNA polymerase II subunits (Fig. 1) and tRNA synthases (S5 Fig.) remained down-regulated in comparison with GS115 after 4h. Not only does this reflect the complete cessation of growth but a significant down-regulation of the potential for *de-novo* protein synthesis.

#### Membrane remodelling

The switch to methylotrophy requires biogenesis of peroxisomes; the location of the first steps in methanol oxidation. In *S*. *cerevisiae PE*X*3* and *PEX19* are essential for early stage biogenesis [34], with Pex3p being secreted into the ER and together with Pex19p being critical for the formation of pre-peroxisomal vesicles [35]. This falls into 2 distinct families in *S*. *cerevisiae*, one containing the RING finger peroxisome membrane proteins (PMPs,) Pex2p, Pex10p and Pex12p, and the other docking PMPs, Pex13p and Pex14p, which are subsequently fused together through the action of Pex1p and Pex6p to form a functional translocon for matrix proteins, with translocation being aided by Pex5p. Expression of the *P*. *pastoris* homologues was strongly induced within 2h of methanol addition in GS115 (S6 Fig. PAS_chr4_0794, chr3_0043, chr4_0759, chr_1073, chr2-1_0715, chr2-2_0207, chr2-2_0186). The expression of *SEC61* in GS115 was down-regulated within 2h of methanol addition (S16 Fig. PAS_chr1-3_0202), which is surprising as the ER translocon formed by Sec61p has been shown to be essential for import of the peroxisomal membrane proteins into the ER. However, due to the transient and long term effects of switching to a poorer substrate peroxisome biogenesis coincides with a starvation response typified by an increase in autophagy (S7 Fig.). Thus, reduction of expression of *SEC61* has to be seen in the light of declining ER capacity. Expression of genes associated with autophagy (S7 Fig.), proteasomal activity (S8 Fig.) and ubiquitin mediated proteolysis (S9 Fig.) were strongly induced in all three strains within the first 2 hours of methanol addition, consistent with a period of extensive membrane remodelling, with some returning to near basal levels within 4h.

#### Energy metabolism and biosynthesis

**Fig 2:**
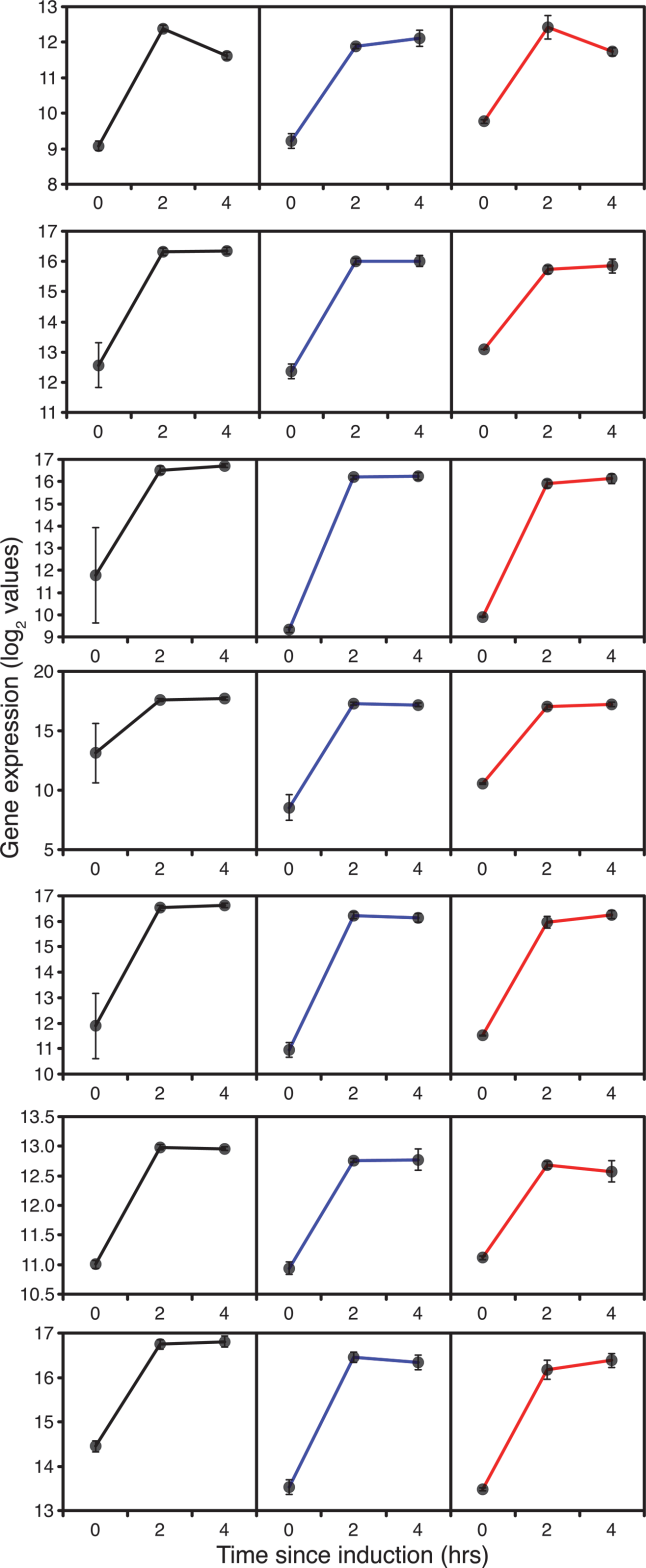
Expression of genes associated with methanol oxidation and assimilation during fed-batch culture, 0 (before), 2 and 4 hours after the start of methanol addition to wild-type GS115 (black), *TRY1-1*(blue) and *TRY1-3* (red), strains containing 1 and 3 gene copies of the human typsinogen gene, respectively, under the control of the *AOX1* promoter. From top to bottom, panels represent *AOX1*, *FDH1*, *FGH1*, *DAS1*, *DAK2*, *TPI1* and *RIB1*. Error bars show SEM.

On top of the expected induction of methanol utilisation gene expression, the transient starvation response, evident from the effects on macromolecule synthesis, was expected to have a significant effect on general energy metabolism. In GS115 and to a lesser extent *TRY1*-1, within the first 2 hours there was an increase in expression of numerous respiratory chain components and the F-type ATPase (S10 Fig.), most of which returned to basal levels of expression after 4h. However, with TRY1-3, most genes retained their 2h expression levels or gradually increased in expression over 4h (eg succinate dehydrogenase/fumarate reductase PAS_chr2−2_0283). Clearly, during this time cells were going through a major reprogramming so, despite the reduction in growth rate, there was an increased energy demand from both methanol and endogenous energy sources. All of the key genes encoding steps in methanol metabolism and assimilation (e.g. *AOX1*, catalase, *FDH*, *DAK2*, *TPI1*) including riboflavin biosynthesis (*RIB1*) showed significant induction over the first 2 hours after methanol addition (Fig. 2) in all strains. *AOX1* expression increased 8 fold between t = 0 and t = 2h. Although *AOX1* expression levels fell slightly between 2 and 4 hours, expression of most of the other genes was maintained at the 2h level indicating that cells should have adapted to growth on methanol by that point.

**Fig 3:**
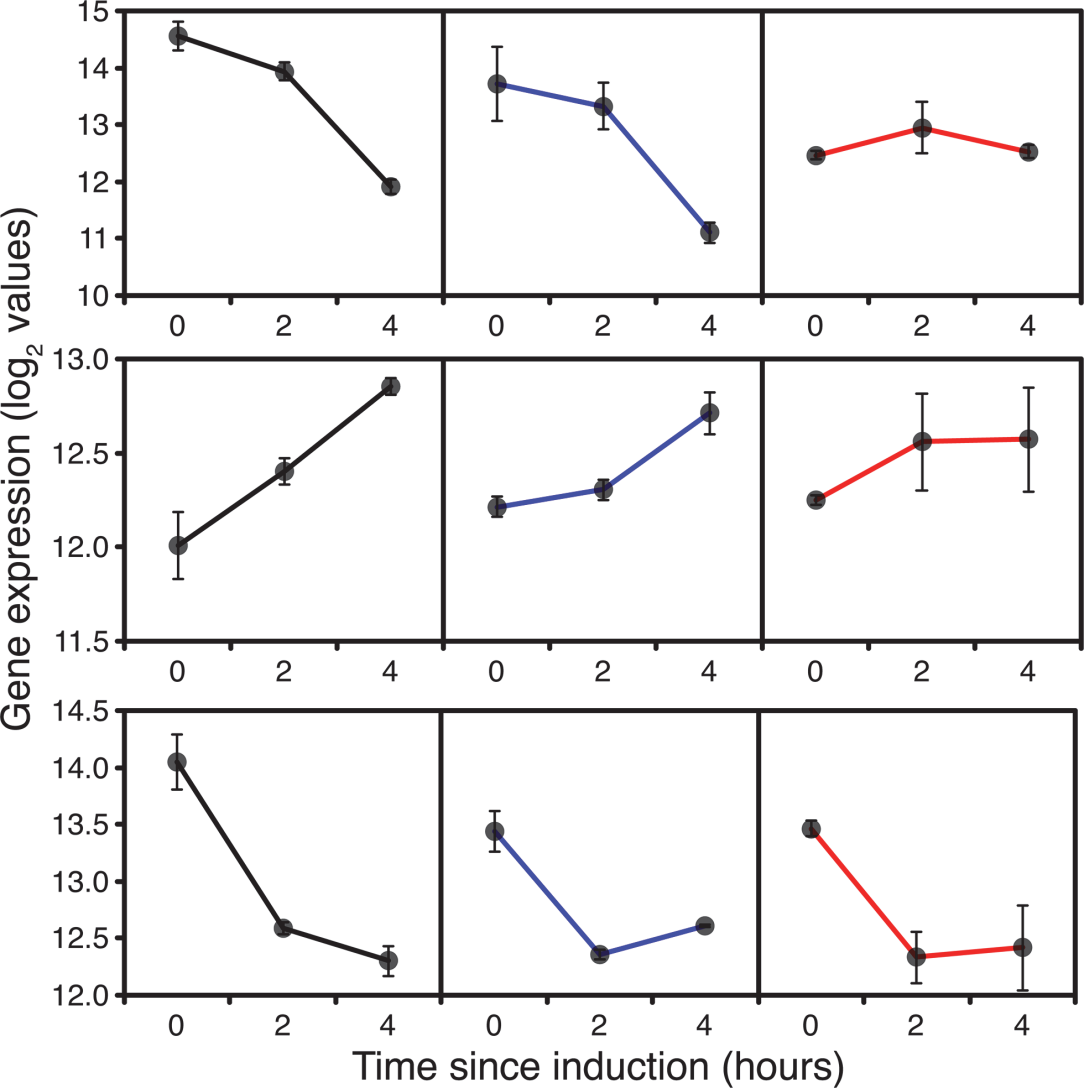
Expression of *PCK1*, *PYC* and *PYK* during fed-batch culture, 0 (before), 2 and 4 hours after the start of methanol addition to wild-type GS115 (black), *TRY1*-1 (blue) and *TRY1*-3 (red). Error bars show SEM.

After 2h expression of genes encoding steps in the TCA and glyoxylate cycles exhibited a combination of up and down regulation in a trend which continued between 2 and 4h (S11 Fig.). This was consistent with the TCA cycle switching from energy generating to biosynthetic mode as would be expected once formaldehyde and formate oxidation had become the major generators of NADH, with down-regulation of expression of pyruvate dehydrogenase and α-ketoglutarate dehydrogenase and upregulation of pyruvate carboxylase, the reversible steps between oxaloacetate and succinate, together with citrate synthase and both mitochondrial and cytosolic NADP^+^ dependent isocitrate dehydrogenases (S11 Fig.). Aconitase and fumarase both showed transient upregulation. Consistent with this pattern, pyruvate kinase and the gluconeogenic phosphoenolpyruvate (PEP) carboxykinase encoded by PCK1 were both down-regulated (Fig. 3).

Glycolysis/gluconeogenesis (S12 Fig.) and pentose phosphate (S13 Fig.) pathways displayed broadly the expected patterns, with upregulation of steps associated with the xylulose monophosphate pathway (XMP) and associated carbon skeleton rearrangements (eg PAS_chr2-2_0337 and PAS_chr3_0868). However, given that the cells were growing on glycerol and the output from the XMP is glyceraldehyde-3-phosphate, significant changes in hexose metabolism were not expected. Therefore, transient induction of hexokinase (PAS_chr1-4_0447, PAS_chr3_1192, PAS_chr1-4_0561 and PAS_chr4_0624), phosphoglucomutase (PAS_chr 1-4_0264), glucose-6-phosphate dehydrogenase (PAS_chr 2-1_0308) suggest that during adaptation to growth on methanol, metabolic reserves of glycogen, trehalose and cell wall β,D glucans (see below) were being degraded. An additional feature was the induction of mitochondrial alcohol (PAS_chr2–1_0472) and aldehyde dehydrogenases (PAS_chr2–2_0853 and PAS_chr3_0987). The former could have been involved in redox shuttling across the mitochondrial membrane [36], while the latter might be supplying acetate for histone acetylation which must have increased following the massive induction of the nuclear acetylCoA synthase 2 (PAS_chr3_0403)

**Fig 4:**
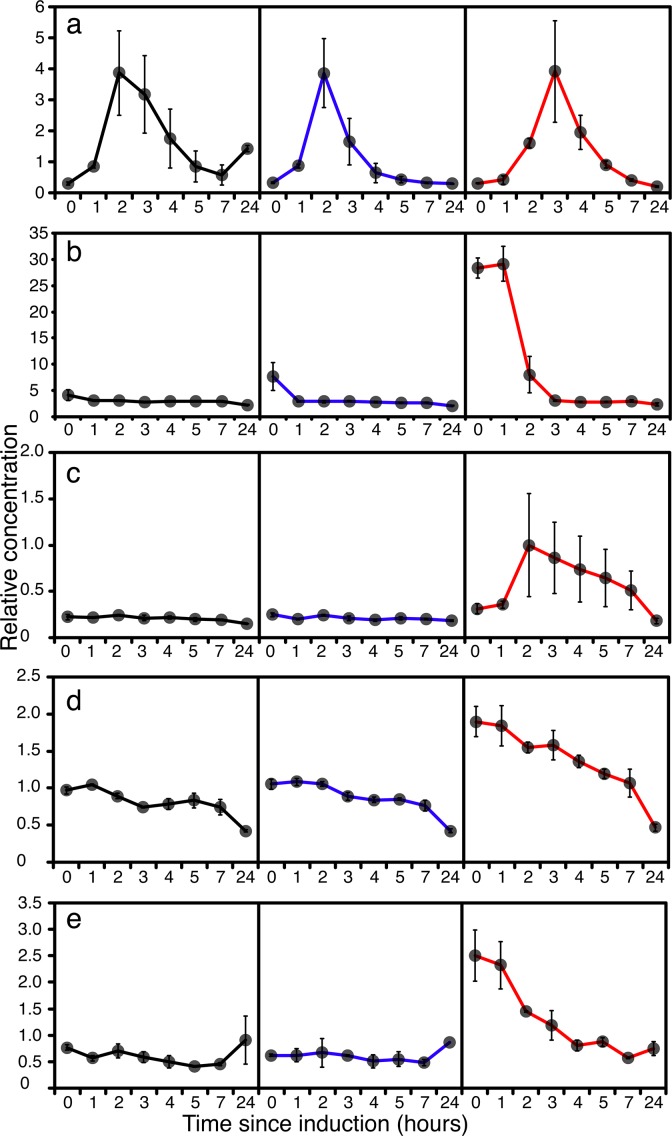
Metabolite accumulation profiles during fed-batch culture from 0 (before) to 24 hours after the start of methanol addition to wild-type GS115 (black), *TRY1-1*(blue) and *TRY1-3* (red), strains containing 1 and 3 gene copies of the human typsinogen gene, respectively, under the control of the *AOX1* promoter. From top to bottom, panels represent a) formate (external), b) arabitol (external), c) trehalose (external), d) α / β-D-glucose (external), e) lactate (internal). Error bars show SEM.

At a metabolomic level, induction of methanol oxidation was characterised by increased extracellular production of 3-hydroxybutyrate and an intracellular and extracellular spike in formate (Fig. 4A), which were more pronounced in GS115 than *TRY1*-1. As the former was not observed with *TRY1*-3, evidence that acetylCoA acetyltransferase activity was enhanced in GS115 compared to the recombinant may be significant. Because metabolite profiles may lag transcriptional induction, the formate spike is difficult to track but the increased expression of S-(hydroxymethyl)glutathione dehydrogenase and S-formyl glutathione hydrolase without increased formate dehydrogenase activity in the 0–2h and 0–4h post-induction profiles (results not shown) suggests that there may have been some imbalance. Taken together, these two profiles suggest a possible redox imbalance in cells adapting to growth on methanol, inhibiting the induction of formate dehydrogenase and using acetoacetate as a redox sink.

However, this transcriptional and metabolomic pattern was different with *TRY1*-3 where, at 4h, glycolysis (S12 and S15 Figs.) and a classic TCA cycle (S11 and S14 Figs.) remained up-regulated compared to GS115. This is consistent with the up-regulation of glycogen breakdown at both 2h and 4h in *TRY1*-3 (compared to GS115), suggesting greater starvation (lack of methanol derived energy). This pattern is also clear from a *TRY1*-3 time course where mobilisation of carbohydrate reserves was evident within 1h of induction. The first 4h of induction after methanol addition were characterised by a transient rise in extracellular α- and/or β-D-glucose (the two mutarotate in solution) accumulation in all strains, but which was particularly pronounced in *TRY1*-3 (Fig. 4D). α-D-glucose production could reflect the breakdown of trehalose, β-D-glucose on the other hand would be derived from β-D-glucans present in the yeast cell wall, indicating an active breakdown process. Between 0 and 1h, the gene encoding β,1,4-D glucanase was significantly induced, consistent with the latter, while at 2h the relevant (α and β) hexokinases (PAS_chr1–4_0447, PAS_chr3_1192) were up-regulated in *TRY1*-3 compared to GS115, indicating that the cells were trying to actively scavenge and import this glucose.

By 4h, both GS115 and *TRY1*-1 were recovering from the initial shock of transfer from glycerol to methanol and tRNA synthases were up-regulated compared to their expression levels at 2h. Comparison with *TRY1*-3 shows that the tRNA synthases were neither significantly upregulated in a 0–4h time course comparison and were significantly down-regulated (or less up-regulated) in a 4h comparison between *TRY1*-3 and GS115 or *TRY1*-1. Thus, this recovery was happening much more slowly in *TRY1*-3, consistent with the delay in the characteristic spike in formate accumulation in *TRY1*-3 cultures (Fig. 4A).

#### Expression associated with heterologous protein production

In *TRY1*-1 and *TRY1*-3 the switch to methanol as a substrate induces heterologous protein production and, as revealed above, this is against a background of cells undergoing transient (in the case of *TRY1*-1) or prolonged starvation. A number of responses associated with the switch from glycerol to methanol could clearly have a significant effect on secretion. In particular, in GS115 there was an increase in expression of ER specific ubiquitin ligase (PAS_chr3_0924) and ER associated degradation (ERAD, PAS_chr1-1_0084), together with a decrease in the expression of some key ER associated protein export proteins such as Sec61 (PAS_chr2-2_210, PAS_chr1-3_0202) and Hsp40 (S16 Fig.). Together with increased proteasomal activity and autophagy, this indicates that the switch to methanol utilisation actually results in degradation and recycling of some of the ER at a time when the demand for heterologous protein secretion is increasing.

Activities specifically induced or down-regulated as a result of heterologous protein expression and secretion were assessed by comparison between the array profiles of *TRY1*-1 or *TRY1*-3 and the parent strain GS115 at 2 and 4h after induction. In GS115 the 0–2h comparison showed down-regulation of some ER related export functions, probably related to the inter-conversion of ER to peroxisomes and reduction in ER capacity. Comparison between *TRY1*-1 and GS115 after 2h induction (S16 and S17 Figs.) shows up-regulation of genes encoding the Sec61 translocon (*SEC61* α and γ, *SEC62* and *SEC63* PAS_chr2–2_210, PAS_chr1-3_0202, PAS_chr3_1014), the ER Kar2p chaperone complex (*KAR2*/*BiP* PAS_chr2–1_0140, *HSP40*) and protein disulphide isomerase (*PDI* PAS_chr4_0844), both key components of the ER protein folding machinery, dolichyl-diphosphooligosaccharide-protein glycosyltransferase (*STT*), oligosaccharide transferase (*OST* PAS_chr2–1_0423, PAS_chr3_0741, PAS_chr4_0610), the glycoprotein chaperone calnexin (*CNX*) together with *SEC23*/*24* (COPII related). The ERAD pathway and proteasome biogenesis were not significantly different from GS115, although both of these pathways were up-regulated between 0 and 2h in GS115. Some heterologous protein expression and secretion related activities were clearly induced at time 0 (see below). Nonetheless, time course comparison of *TRY1*-1 between 0 and 2h shows that *SEC61*, *OST*, *CNX* and *PDI* were also further up-regulated by induction of *TRY1*-1 expression, compared to GS115; after 4h induction, the pattern was clearly retained, with no relative up-regulation of the ERAD pathway.

**Fig 5:**
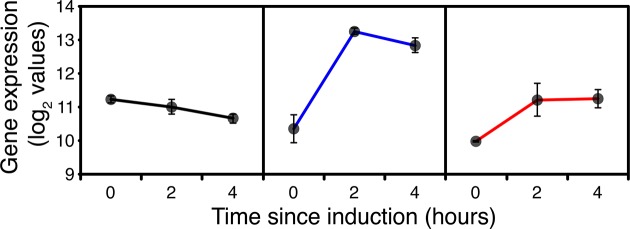
Expression of *HAC1* during fed-batch culture, 0 (before), 2 and 4 hours after the start of methanol addition to wild-type GS115 (black), *TRY1-1*(blue) and *TRY1-3* (red), strains containing 1 and 3 gene copies of the human typsinogen gene, respectively, under the control of the *AOX1* promoter. Error bars show SEM.

Induction of the unfolded protein response, characterised by increased expression of the chaperones Kar2p and Pdi is controlled by the production of Hac1p, the product of a spliced *HAC1* mRNA. In *P*. *pastoris*, control of Hac1p expression appears to be via up-regulation of *HAC1* expression rather than control of the splicing reaction [28] Consistent with this, the expression of *HAC1* increased approximately 5 fold in the first 2h of induction of *TRY1*-1, whereas it barely changed in GS115 (Fig. 5). Significantly, the lack of further induction of ERAD suggests that, with a single copy of *TRY1*, the increased traffic through the ER is managed adequately.

Based on the tube culture results we would have expected that the ER related chaperones would have been further up-regulated in *TRY1*-3 compared to the situation with *TRY1*-1 at 2h, and ERAD-related activities would also have been stimulated. Surprisingly, although both sets of activities were elevated compared to GS115 at the same time points, they were significantly lower than in *TRY1*-1. Significantly, this lack of induction of UPR in *TRY1*-3 correlated with a relatively minor increase in expression of *HAC1*, compared to that observed with *TRY1*-1, indicating that the complete cessation of growth was not the result of secretion stress (Fig. 5). One of the few secretion related functions that was up-regulated in *TRY1*-3 compared to *TRY1*-1 was *GLCII*, the product of which catalyses both glycosylation and deglycosylation, so could have a scavenging function.

#### Evidence of metabolic stress in *TRY1*-3

**Fig 6:**
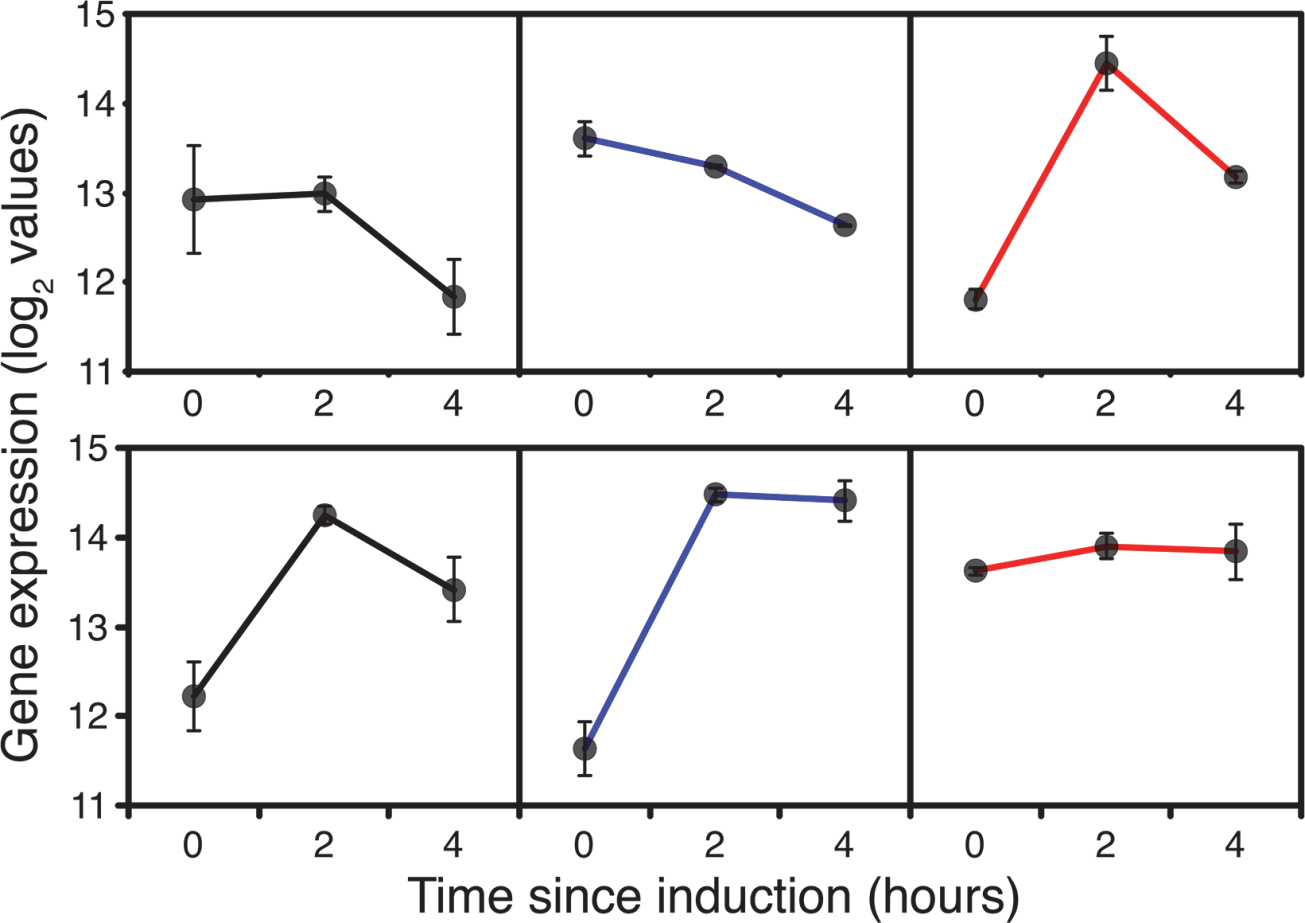
Expression of the gene encoding D-arabitol-2-dehydrogenase (top) and “*YDL124W”* (bottom), 0 (before), 2 and 4 hours after the start of methanol addition to wild-type GS115 (black), *TRY1-1*(blue) and *TRY1-3* (red), strains containing 1 and 3 gene copies of the human typsinogen gene, respectively, under the control of the *AOX1* promoter. Error bars show SEM.

Even before detailed analysis of the microarray data it was clear that *TRY1*-3 cells were behaving differently from both GS115 and *TRY1*-1 cells. Notably, the switch to methanol led to a complete cessation of growth for an extended period and the metabolomics profile showed an initial accumulation of extracellular arabitol, followed by trehalose accumulation (Fig. 4B,C), both signs of metabolic, particularly oxidative stress. Arabitol production derives from reduction of intermediates in the pentose phosphate pathway, such as xylulose or ribulose. The only gene directly identifiable as catalysing this function was "D-arabitol-2-dehydrogenase”, which is clearly strongly induced in *TRY1*-3. However, the timescale of induction (Fig. 6) suggests that it may be responding to the presence of arabitol and be involved in conversion back to ribulose (note that production and uptake are catalysed by different enzymes in *C*. *albicans* [37]). Baumann *et al* [38] suggested that arabitol production was due to the activity of “YDL124W”, which is certainly expressed at a higher level in *TRY1*-3 than the other strains at 0h (Fig. 6). However, the subsequent induction of this activity in GS115 and *TRY1*-1 does not coincide with increased arabitol production in these strains, so the origin of arabitol accumulation in *P*. *pastoris* remains uncertain.

### Expression prior to induction with methanol

Excess glycerol is a catabolite repressor of *AOX1* expression but induction has been observed when methanol was added to cultures growing on limiting glycerol concentrations [39]. Furthermore, a low level (compared to fully induced) of *AOX1* de-repression has been observed when cells are starved of a carbon source and therefore reliant on breakdown of endogenous reserves, and also in glycerol limited continuous cultures [40]. While methanol utilisation pathways genes were clearly induced after addition of methanol, it is apparent (Fig. 2) that all of these steps were being expressed prior to the addition of methanol in GS115 and the recombinant cells.

Significantly, in the context of de-repression of *AOX1* expression, both *TRY1*-1 and *TRY1*-3 showed up-regulation of both cytoplasmic and ER lumen based protein glycosylation activities associated with core N-glycosylation (S17 Fig. eg PAS_c121_0002, PAS_chr3_0944), consistent with the expression of a glycosylated heterologous protein. So, at this stage it was clear that *TRY1* was being expressed, seemingly in a manner which reflected gene copy number (eg PAS_chr3_0944). However, this clearly had a cost to the host cell. Compared to GS115, *TRY1*-1 showed some down-regulation of ribosomal protein synthesis and ribosomal biogenesis, whereas both of these were dramatically down-regulated (S1 Fig.) in *TRY1*-3 (the difference was also evident in a comparison of *TRY1*-1 and *TRY1*-3). In addition *TRY1*-3 had reduced expression of RNA polymerase I and III, pol III associated tRNA 5’ and 3’ end cap processing, purine and pyrimidine biosynthesis, DNA replication and translation initiation factors, and even showed an increase in autophagy (S7 Fig.), ubiquitin ligase (S9 Fig.) and proteasomal activity (S8 Fig.), in comparison with GS115. Increased autophagy and proteasomal activities are similar to the pattern seen when comparing induction in GS115 between 0 and 2h. However, while at t = 0, there may also have been some peroxisome biogenesis associated with the depression of methanol oxidation genes, nutritional starvation responses/growth rate reduction was also greater in *TRY1*-3 than GS115.

## Discussion

The *AOX1* induction system is described as tightly controlled and dependent on methanol for induction but the level of control is substrate and condition dependent [39]. While excess glycerol is a strong catabolite repressor, starvation or limiting concentrations of glycerol allow de-repression of *AOX1* gene expression. This means that it is possible to obtain growth in continuous culture on glycerol-methanol or glucose-methanol combinations so long as the multi-carbon substrate is in limiting amounts [40]. In a typical induction regime, during the early stage of fed-batch growth on glycerol, the glycerol concentration in the culture will be high, but as the cell density increases, just before induction with methanol the glycerol concentration will have become growth limiting (1) (ie the concentration in the culture will be very low). Thus, it is not surprising that heterologous protein expression will have started prior to the addition of methanol. With the multiple gene-copy strains it is possible that de-repression occurs even earlier due to the change in stoichiometry of repressors and cognate binding site in the *AOX1* promoter.

De-repression of heterologous protein synthesis prior to the switch to methanol clearly presents an additional metabolic load on the cells, which are already undergoing a decline in growth rate in a fed-batch regime with a constant supply of a growth limiting substrate. Comparison between GS115, *TRY1*-1 and *TRY1*-3 at t = 0 confirms that heterologous protein expression was accompanied by a typical starvation related down-regulation of ribosomal activity and up-regulation of autophagy and proteasomal activity in a *TRY1* copy-number-dependent pattern. It should be recognised that this scenario is fundamentally different from a typical tube or shake flask culture where cells are grown in an excess of an initial carbon source, harvested and then transferred into a methanol medium.

The switch from glycerol to methanol clearly has further dramatic effects on growth rate, metabolic and energetic priorities of the cells and internal membrane organisation. While the expression of a single copy of *TRY1* increased this burden, it was tolerated and in many ways exhibited a classical controlled UPR transcriptional profile, with little further induction of ERAD, although notably the level of *HAC1* induction (Fig. 4) was higher in fed-batch than in the tube culture experiments. However, the presence of 3 copies of *TRY1*, which gave a severe UPR response and reduced protein secretion in tube cultures, presumably due to the effects of ERAD [6], had a completely different profile in methanol-induced fed-batch culture. A high level of *AOX1* induction accompanied by a low level of *HAC1* induction indicates a disconnection between transcription of *TRY1* (under the control of the *AOX1* promoter) and protein export. The most likely explanation for this is some form of translational arrest meaning that in *TRY1*-3 the *TRY1* transcripts were not being quantitatively translated and therefore traffic through the ER was significantly less than with *TRY1*-1. This is consistent with the observation of similar levels of induction of the methanol dissimilation pathway genes in *TRY1*-3 and *TRY1*-1, accompanied by transcriptional and metabolomic evidence for extensive breakdown of carbohydrate reserves and slow adaptation of *TRY1*-3 to grow on methanol.

Translational arrest can occur due to a variety of stress factors including high levels of UPR [41], heat shock [42], nutritional [43] and osmotic stress [44]. In the case of *TRY1*-3, high levels of UPR were clearly not the cause, and differences in heterologous protein expression were unlikely to cause osmotic stress. Stress-related translational arrest occurs through interference with one of two stages of the assembly of a functioning ribosomal translation initiation complex. This leads to the formation of stress bodies which sequester specific mRNAs and certain initiation factors meaning that only a selective subclass of mRNAs can be translated [42, 45]. Several stress factors stimulate Gcn2p to phosphorylate eIF2 α which inhibits the GTP binding necessary for translational initiation. A later stage in initiation complex assembly is dependent on a functional TOR complex, which responds to the nutritional status of the cell. Under general nutritional starvation the function of Tor2p is compromised which indirectly leads to the degradation of eIF-4G [46], which normally serves as a scaffold for late stage assembly of the translation initiation complex.

**Fig 7:**
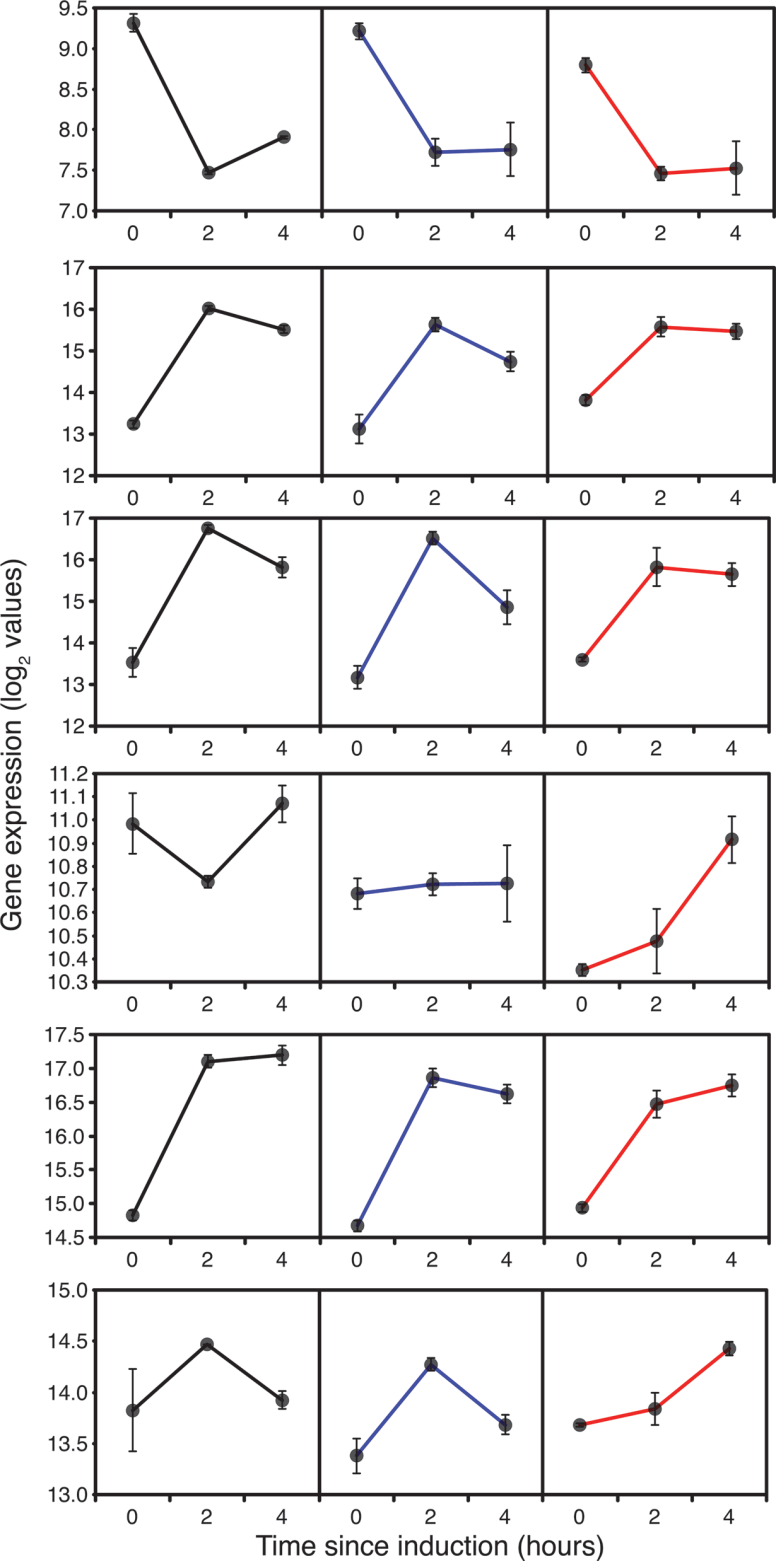
Expression of starvation and stress response genes, 0 (before), 2 and 4 hours after the start of methanol addition to wild-type GS115 (black), *TRY1-1*(blue) and *TRY1-3* (red), strains containing 1 and 3 gene copies of the human typsinogen gene, respectively, under the control of the *AOX1* promoter. From top to bottom, panels represent *GCN4*, *TRX2*, *TSA1*, *AHP1*, *SOD1*, *SOD2*. Error bars show SEM.

The most likely stress factor which could give rise to translational arrest was a nutritional imbalance resulting from the demands for a high level of protein synthesis in cells undergoing nutritional limitation. It is evident that the TOR system must have been responding to general starvation in GS115 and the *TRY1* constructs, as this is the master repressor of autophagy [47] and also indirectly controls ribosomal protein production [48]. However, if translational arrest occurred in GS115 and *TRY1*-1 it was short lived, compared to the situation with *TRY1*-3, which indicates that other factors may have been involved. Given the higher demand for protein synthesis in *TRY1*-3, it was possible that, combined with general starvation, this led to an effective starvation for amino acids (ie demand outstripped supply). This has a dedicated control mechanism (general amino acid control responding to the presence of uncharged tRNAs) linked to phosphorylation of Gcn2p but also involving the (degree of starvation-dependent) control of translation of Gcn4p, a transcriptional activator of amino acid biosynthetic genes [49]. Although the activation of this pathway involves phosphorylation and translational control (undetectable in a microarray), there was no evidence for higher levels of expression of amino acid biosynthetic genes in *TRY1*-3 compared to GS115 at any of the time points. Indeed, the transcriptional abundance of *GCN4* in *TRY1*-3 at t = 0 was significantly below that of GS115 and *TRY1*-1, suggesting that whatever stress was leading to translational arrest was also limiting the possibility for activation of the *GCN4* dependent signal transduction pathway (Fig. 7). Interestingly, the profile of *GCN4* expression reflects the changes in growth rate, with a reduction between 0 and 2h followed by an increase at 4h in both GS115 and *TRY1*-1, whereas in *TRY1*-3 that expression level stays low from 0–4h.

Major clues as to the likely cause of translational arrest come from the metabolomic profile of *TRY1*-3, with a spike of intracellular and extracellular arabitol between 0–4h being consistent with cells being stressed either before or during induction with methanol. Yeasts characteristically produce polyols such as glycerol and arabitol [50] in response to a variety of environmental stresses (osmotic, temperature, oxidative). In *S*. *cerevisiae*, glycerol appears to be the major stress-related polyol, with arabitol playing a minor role, whereas in *P*. *pastoris* and in *P*. *anomala* the predominant polyol appears to be arabitol [51, 52]. In the closely related *Candida* spp, glycerol is produced in response to osmotic stress, whereas production of arabitol results from either temperature stress or oxidative stress [53]. In our case, oxidative stress was unlikely to be the cause given that *TRY1*-3 showed a lower induction of respiratory activity 2h after methanol addition compared to GS115. Additionally, oxidative stress has characteristic transcriptional induction signatures [54], with hydrogen peroxide (eg *TRX2*, *TRR1*, *TSA1* and *CTT*) and organic peroxides inducing expression of *AHP1* and *GPX1-3* and superoxide inducing expression of *SOD1*, *SOD2*. While many of these activities were induced as cells adapted to growth on methanol there was no significant difference between *TRY1*-1 and *TRY1*-3 in any of these signatures (Fig. 7), suggesting that they were managed, rather than stress responses.

Even before methanol addition *TRY1*-3 had elevated levels of intracellular lactate (Fig. 3E) which suggests a) that glycogen derived glucose was already a significant energy source (lactate production from glycerol would have no metabolic advantage) but b) there was an imbalance between the NADH generation in the cytosol and transfer or use in the mitochondria. Combined with evidence for a restricted increase in respiratory activity after methanol addition (compared to GS115), the production of arabitol can be seen, not as a general stress response, but as a response to redox imbalance in the cytosol. In *S*. *cerevisiae*, cytoplasmic redox stress is known to induce glycerol production in a pathway which is regulated separately from the general stress response pathway [55]. Even though a high dissolved oxygen tension was maintained through control of aeration, limitation of mitochondrial capacity to exchange reducing equivalents with the cytosol would have the same physiological consequences as hypoxia, in which arabitol production has also been observed [38]. Similarly, the filamentous fungus *Aspergillus niger* produces large amounts of polyols, principally mannitol, in response to low oxygen levels, to oxidize the NADH that would otherwise accumulate as a consequence of reduced oxidative metabolism [56, 57]. Given that the de-repressed expression of *TRY1* appeared to be following the expected profile based on copy number, this suggests that the high metabolic demands of heterologous protein production in the *TRY1*-3 strain, combined with the metabolic stress of a classical glycerol to methanol fed-batch regime resulted in a severe cytosolic-mitochondrial redox imbalance, sufficient to cause a stress response which appears to have resulted in translational arrest. While the effects of redox stress on polyol production have previously been demonstrated in both *S*. *cerevisiae* and *P*. *pastoris*, this has not previously been characterised as a major stress response that can lead to translational arrest. In *S*. *cerevisiae* this may not have been observed because, as a Crabtree-positive yeast, physiologically relevant redox stress can be resolved by the production of ethanol.

Examination of the metabolomic profile of all of the strains suggests that a degree of redox stress is characteristic of the switch from glycerol to methanol even in the absence of heterologous protein synthesis. All strains start to accumulate some arabitol and β-hydroxybutyrate (less in *TRY1*-3 until 4h after induction) after the addition of methanol and also have a characteristic formate spike, where formate accumulates transiently, presumably due to a redox imbalance reducing the rate of formate oxidation. However, in *TRY1*-3 arabitol accumulation is higher, the formate spike appears later and only in this strain is there a significant production of α, α trehalose, the production of which coincides with the peak of arabitol production. Trehalose is a classic stress response molecule which can have a number of functions including providing protection of proteins and membranes after heat shock and control of glycolysis. Intracellular accumulation appears to be the result of an imbalance between synthesis and degradation, resulting primarily from post-translational control of the different activities.

A clear and unexpected pattern has emerged from this data which provides an additional explanation for poor secreted heterologous protein production in bioreactors, where results sometimes differ from those obtained in shake flasks. The standard fed-batch production regime leads to de-repression of heterologous protein production before methanol induction, and this is in a background of metabolic stress caused by declining growth rate. With the addition of methanol, cells with a high existing metabolic load due to heterologous protein production undergo a stress response caused by a redox imbalance between the cytosol and mitochondria. This causes a transient and possibly selective translational arrest, which affects both heterologous protein production and the rate of adaptation to growth on methanol. As a result, titres of heterologous proteins produced using this standard fed-batch production regime, may be dramatically lower than those predicted from shake flask studies.

## Supporting Information

S1 Fig Expression levels of Try1p in tube cultures. Try1p levels (arbitrary scale) in the culture supernatant of 50ml tube cultures of recombinant strains containing 1, 2 and 3 copies of the *TRY1* grown for 24h in BMMY analysed on an Experion Pro260 protein analysis chip (BioRad). The same volume of supernatant was applied to each lane.
(TIF)

S2 Fig Expression levels of ribosome biogenesis genes. Log 2 normalised expression levels of ribosome biogenesis genes grouped according to KEGG pathways. Genes are uniquely identified by PAS (PAStoris) codes and expression was determined at 0, 2 and 4h after methanol addition in fed-batch cultures of GS115, TRY1-1 and TRY1-3.The associated trees cluster genes with similar expression profiles across all conditions.
(PDF)

S3 Fig Expression levels of ribosomal protein biosynthesis genes. Log 2 normalised expression levels of ribosomal protein biosynthesis genes grouped according to KEGG pathways. Genes are uniquely identified by PAS (PAStoris) codes and expression was determined at 0, 2 and 4h after methanol addition in fed-batch cultures of GS115, TRY1-1 and TRY1-3.The associated trees cluster genes with similar expression profiles across all conditions.
(PDF)

S4 Fig Expression levels of genes encoding RNA Polymerase I, II and III associated proteins. Heat maps of log 2 normalised expression levels of genes encoding RNA Polymerase I, II and III associated proteins grouped according to KEGG pathways. Genes are uniquely identified by PAS (PAStoris) codes and expression was determined at 0, 2 and 4h after methanol addition in fed-batch cultures of GS115, TRY1-1 and TRY1-3.The associated trees cluster genes with similar expression profiles across all conditions.
(PDF)

S5 Fig Expression levels of tRNA synthase genes. Heat maps of log 2 normalised expression levels of tRNA synthase genes. Genes are uniquely identified by PAS (PAStoris) codes and expression was determined at 0, 2 and 4h after methanol addition in fed-batch cultures of GS115, TRY1-1 and TRY1-3.The associated trees cluster genes with similar expression profiles across all conditions.
(PDF)

S6 Fig Expression levels of genes encoding proteins involved in peroxisomal biogenesis. Heat maps of log 2 normalised expression levels of genes encoding proteins involved in peroxisomal biogenesis grouped according to KEGG pathways. Genes are uniquely identified by PAS (PAStoris) codes and expression was determined at 0, 2 and 4h after methanol addition in fed-batch cultures of GS115, TRY1-1 and TRY1-3.The associated trees cluster genes with similar expression profiles across all conditions.
(PDF)

S7 Fig Expression levels of genes associated with autophagy. Heat maps of log 2 normalised expression levels of genes associated with autophagy, grouped according to KEGG pathways. Genes are uniquely identified by PAS (PAStoris) codes and expression was determined at 0, 2 and 4h after methanol addition in fed-batch cultures of GS115, TRY1-1 and TRY1-3.The associated trees cluster genes with similar expression profiles across all conditions.
(PDF)

S8 Fig Expression levels of genes associated with proteasomal activity. Heat maps of log 2 normalised expression levels of genes associated with proteasomal activity, grouped according to KEGG pathways. Genes are uniquely identified by PAS (PAStoris) codes and expression was determined at 0, 2 and 4h after methanol addition in fed-batch cultures of GS115, TRY1-1 and TRY1-3.The associated trees cluster genes with similar expression profiles across all conditions.
(PDF)

S9 Fig Expression levels of genes associated with ubiquitin mediated proteolysis. Heat maps of log 2 normalised expression levels of genes associated with ubiquitin mediated proteolysis, grouped according to KEGG pathways. Genes are uniquely identified by PAS (PAStoris) codes and expression was determined at 0, 2 and 4h after methanol addition in fed-batch cultures of GS115, TRY1-1 and TRY1-3.The associated trees cluster genes with similar expression profiles across all conditions.
(PDF)

S10 Fig Expression levels of genes encoding proteins involved in oxidative phosphorylation. Heat maps of log 2 normalised expression levels of genes encoding proteins involved in oxidative phosphorylation, defined by KEGG families, at 2h and 4h after methanol addition in fed-batch cultures of GS115, TRY1-1 and TRY1-3, compared to those of GS115 before methanol addition. The associated trees cluster genes with similar expression profiles across all conditions.
(PDF)

S11 Fig Expression levels of genes encoding proteins involved in TCA Cycle. Heat maps of log 2 normalised expression levels of genes encoding proteins involved in the TCA Cycle, defined by KEGG families, at 2h and 4h after methanol addition in fed-batch cultures of GS115, TRY1-1 and TRY1-3, compared to those of GS115 before methanol addition. The associated trees cluster genes with similar expression profiles across all conditions.
(PDF)

S12 Fig Expression levels of genes encoding proteins involved in Glycolysis/gluconeogenesis. Heat maps of log 2 normalised expression levels of genes encoding proteins involved in glycolysis/gluconeogenesis, defined by KEGG families, at 2h and 4h after methanol addition in fed-batch cultures of GS115, TRY1-1 and TRY1-3, compared to those of GS115 before methanol addition. The associated trees cluster genes with similar expression profiles across all conditions.
(PDF)

S13 Fig Expression levels of genes encoding proteins involved in pentose phosphate cycle. Heat maps of log 2 normalised expression levels of genes encoding proteins involved in the pentose phosphate cycle, defined by KEGG families, at 2h and 4h after methanol addition in fed-batch cultures of GS115, TRY1-1 and TRY1-3, compared to those of GS115 before methanol addition. The associated trees cluster genes with similar expression profiles across all conditions.
(PDF)

S14 Fig KOBAS comparisons between TCA cycle gene expression levels in *TRY1*–3 and GS115, 4 hours after addition of methanol. Comparisons are based on KEGG pathways. Genes where expression is significantly up-regulated (red) or down-regulated (blue) in *TRY1*-3 compared to GS115 are highlighted. *P pastoris* genes where expression is not significantly different are highlighted in green.
(TIF)

S15 Fig KOBAS comparisons between glycolysis/gluconeogenesis gene expression levels in *TRY1*-3 and GS115, 4 hours after addition of methanol. Comparisons are based on KEGG pathways. Genes where expression is significantly up-regulated (red) or down-regulated (blue) in *TRY1*-3 compared to GS115 are highlighted. *P pastoris* genes where expression is not significantly different are highlighted in green
(TIF)

S16 Fig Expression levels of genes associated with protein processing in the ER. Heat maps of log 2 normalised expression levels of genes associated with protein processing in the ER, grouped according to KEGG pathways. Genes are uniquely identified by PAS (PAStoris) codes and expression was determined at 0, 2 and 4h after methanol addition in fed-batch cultures of GS115, TRY1-1 and TRY1-3. The associated trees cluster genes with similar expression profiles across all conditions.
(PDF)

S17 Fig Expression levels of genes associated with N-glycan biosynthesis. Heat maps of log 2 normalised expression levels of genes associated with N-glycan biosynthesis, grouped according to KEGG pathways. Genes are uniquely identified by PAS (PAStoris) codes and expression was determined at 0, 2 and 4h after methanol addition in fed-batch cultures of GS115, TRY1-1 and TRY1-3. The associated trees cluster genes with similar expression profiles across all conditions.
(PDF)
