# Supplementary figures and images for: Translational Arrest Due to Cytoplasmic Redox Stress Delays Adaptation to Growth on Methanol and Heterologous Protein Expression in a Typical Fed-Batch Culture of Pichia pastoris
Source: PLoS One. 2015 Mar 18;10(3):e0119637. doi: 10.1371/journal.pone.0119637 (PMC4364781; doi:10.1371/journal.pone.0119637)

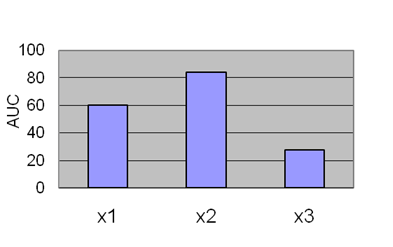

Supplement: S1 Fig — Try1p levels (arbitrary scale) in the culture supernatant of 50ml tube cultures of recombinant strains containing 1, 2 and 3 copies of the TRY1 grown for 24h in BMMY analysed on an Experion Pro260 protein analysis chip (BioRad). The same volume of supernatant was applied to each lane. (TIF) [file pone.0119637.s001.tif]

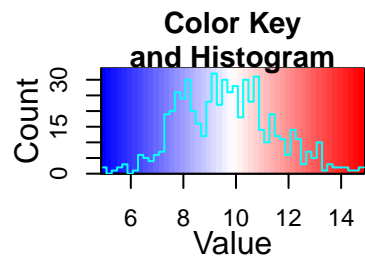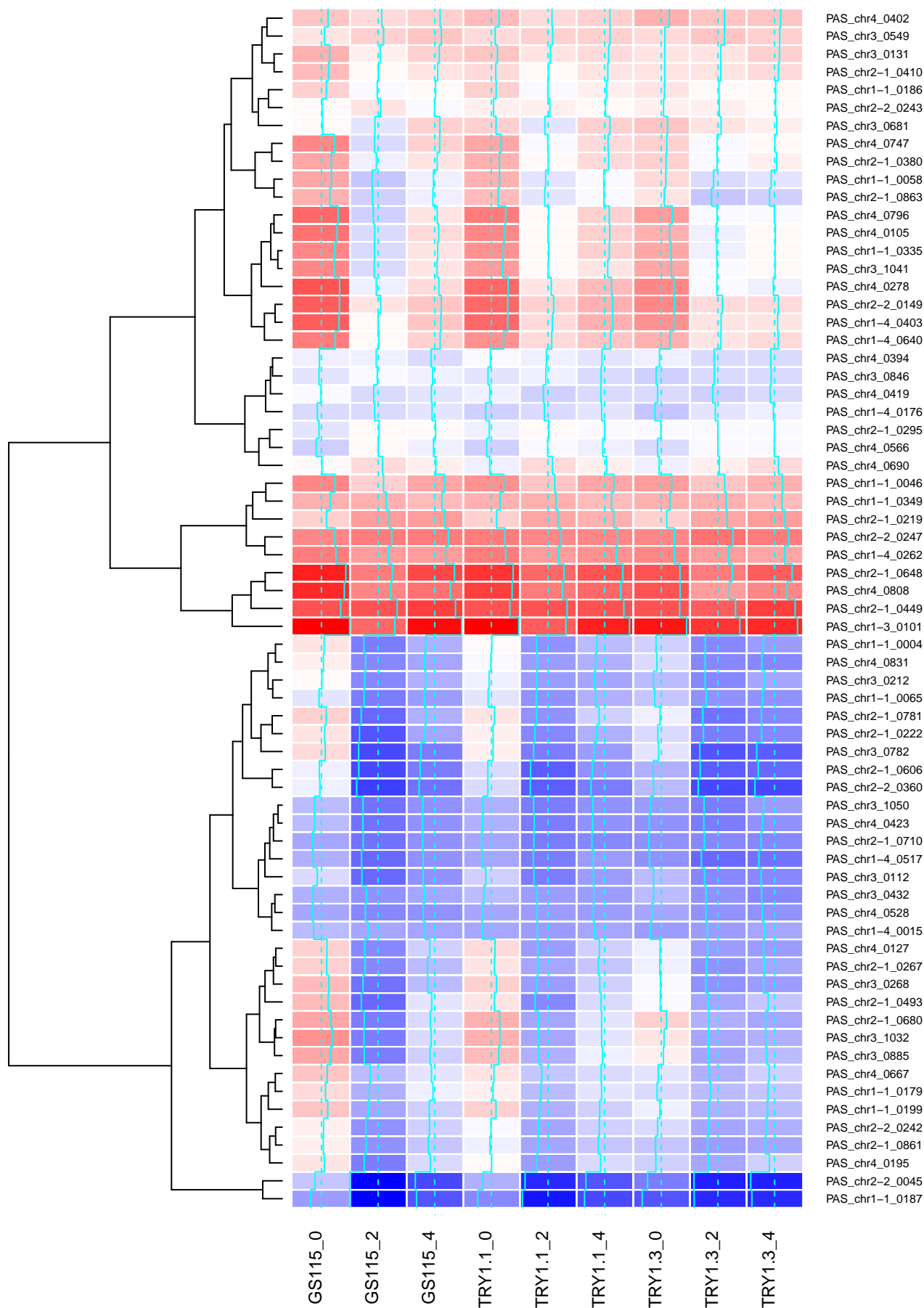

Supplement: S2 Fig — Log 2 normalised expression levels of ribosome biogenesis genes grouped according to KEGG pathways. Genes are uniquely identified by PAS (PAStoris) codes and expression was determined at 0, 2 and 4h after methanol addition in fed-batch cultures of GS115, TRY1-1 and TRY1-3.The associated trees cluster genes with similar expression profiles across all conditions. (PDF) [file pone.0119637.s002.pdf]

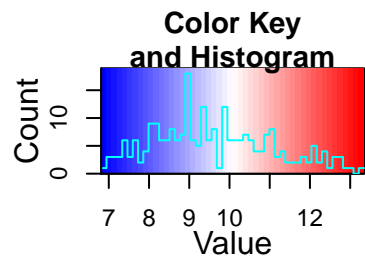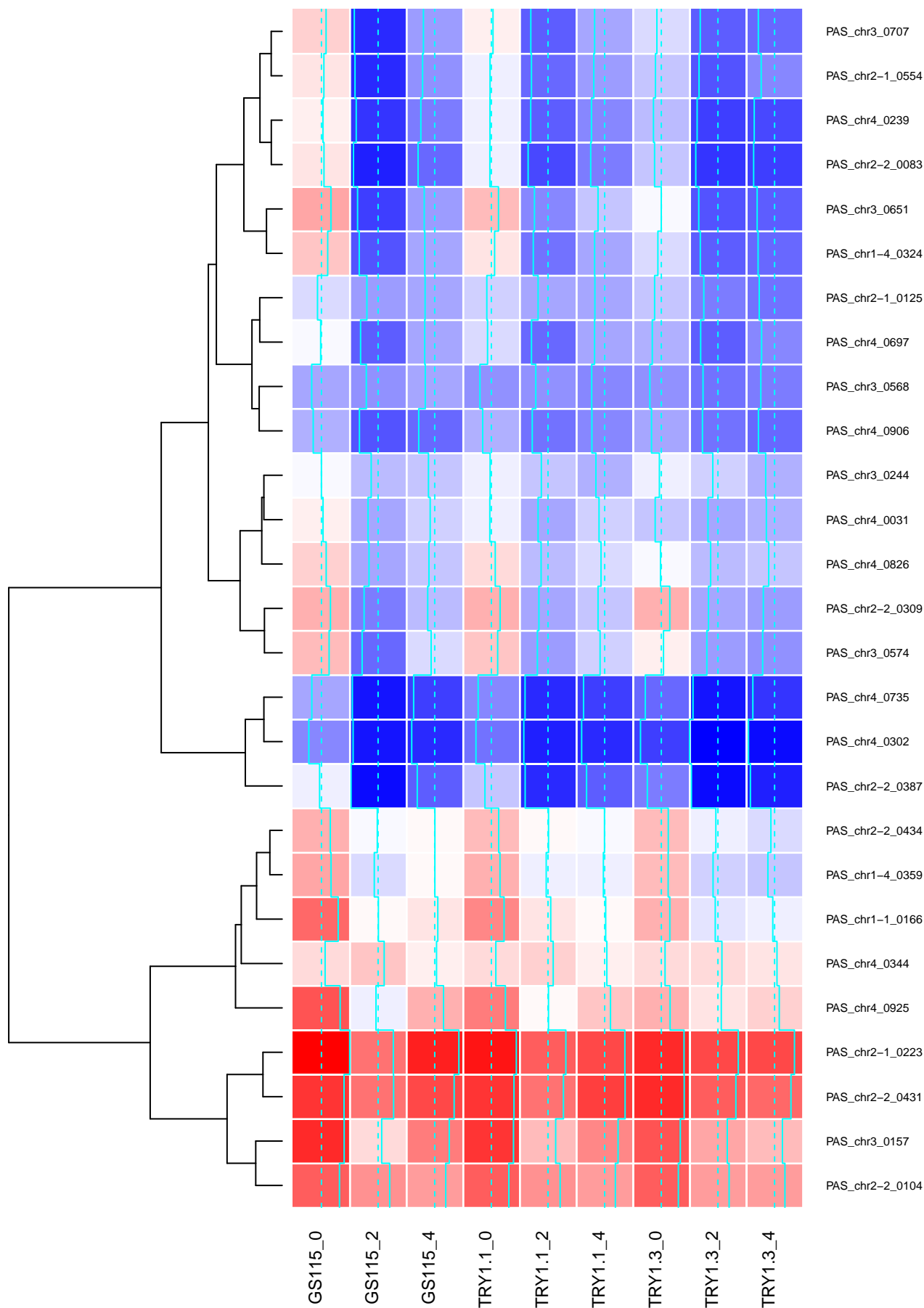

Supplement: S4 Fig — Heat maps of log 2 normalised expression levels of genes encoding RNA Polymerase I, II and III associated proteins grouped according to KEGG pathways. Genes are uniquely identified by PAS (PAStoris) codes and expression was determined at 0, 2 and 4h after methanol addition in fed-batch cultures of GS115, TRY1-1 and TRY1-3.The associated trees cluster genes with similar expression profiles across all conditions. (PDF) [file pone.0119637.s004.pdf]

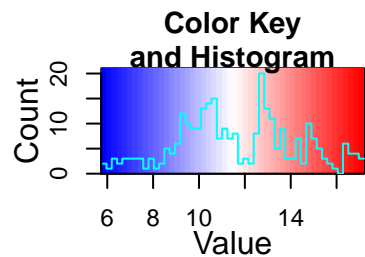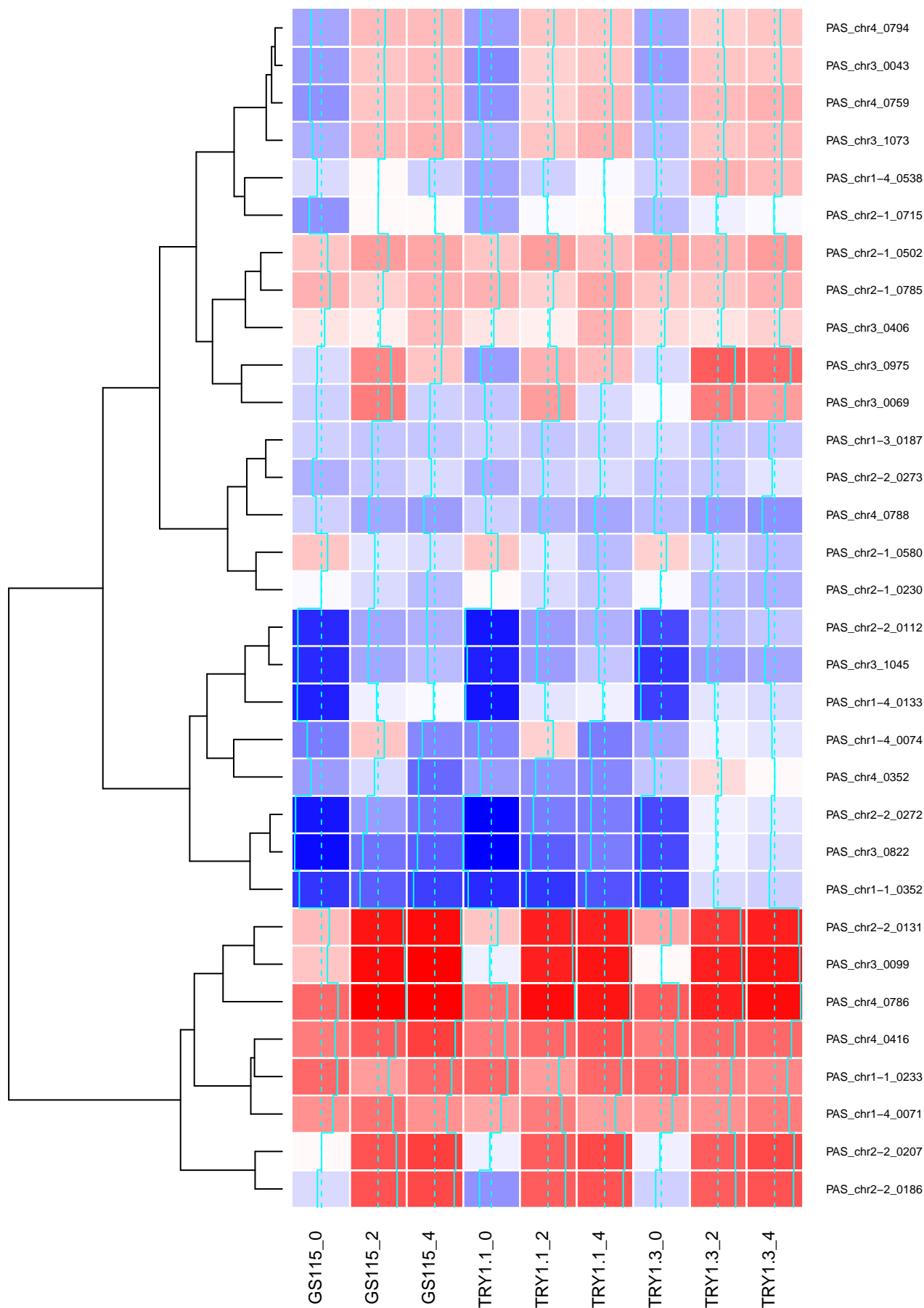

Supplement: S6 Fig — Heat maps of log 2 normalised expression levels of genes encoding proteins involved in peroxisomal biogenesis grouped according to KEGG pathways. Genes are uniquely identified by PAS (PAStoris) codes and expression was determined at 0, 2 and 4h after methanol addition in fed-batch cultures of GS115, TRY1-1 and TRY1-3.The associated trees cluster genes with similar expression profiles across all conditions. (PDF) [file pone.0119637.s006.pdf]

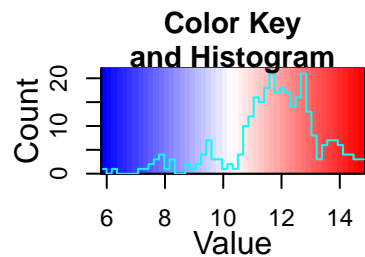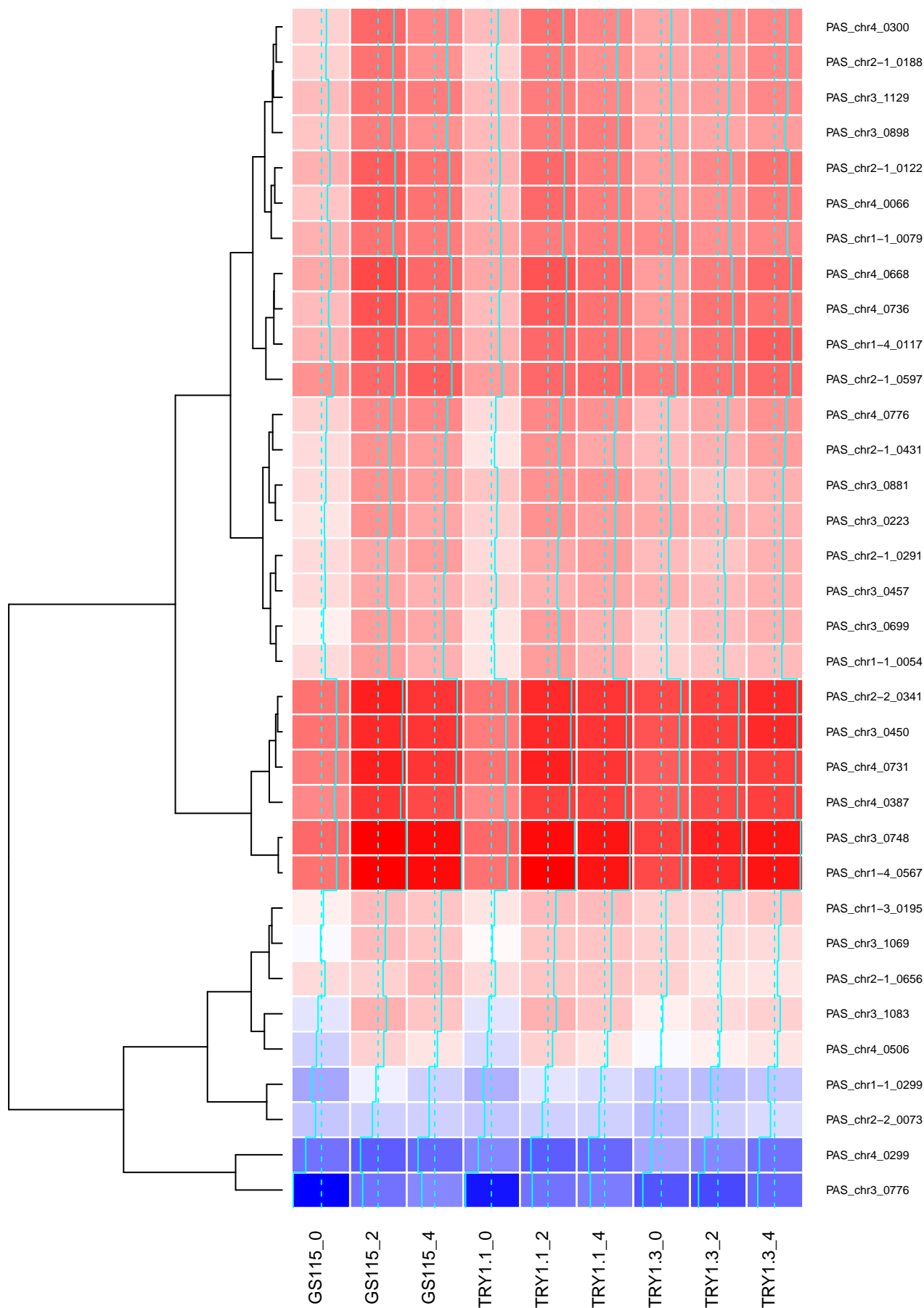

Supplement: S8 Fig — Heat maps of log 2 normalised expression levels of genes associated with proteasomal activity, grouped according to KEGG pathways. Genes are uniquely identified by PAS (PAStoris) codes and expression was determined at 0, 2 and 4h after methanol addition in fed-batch cultures of GS115, TRY1-1 and TRY1-3.The associated trees cluster genes with similar expression profiles across all conditions. (PDF) [file pone.0119637.s008.pdf]

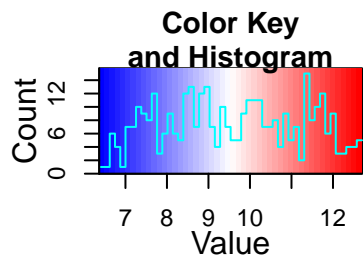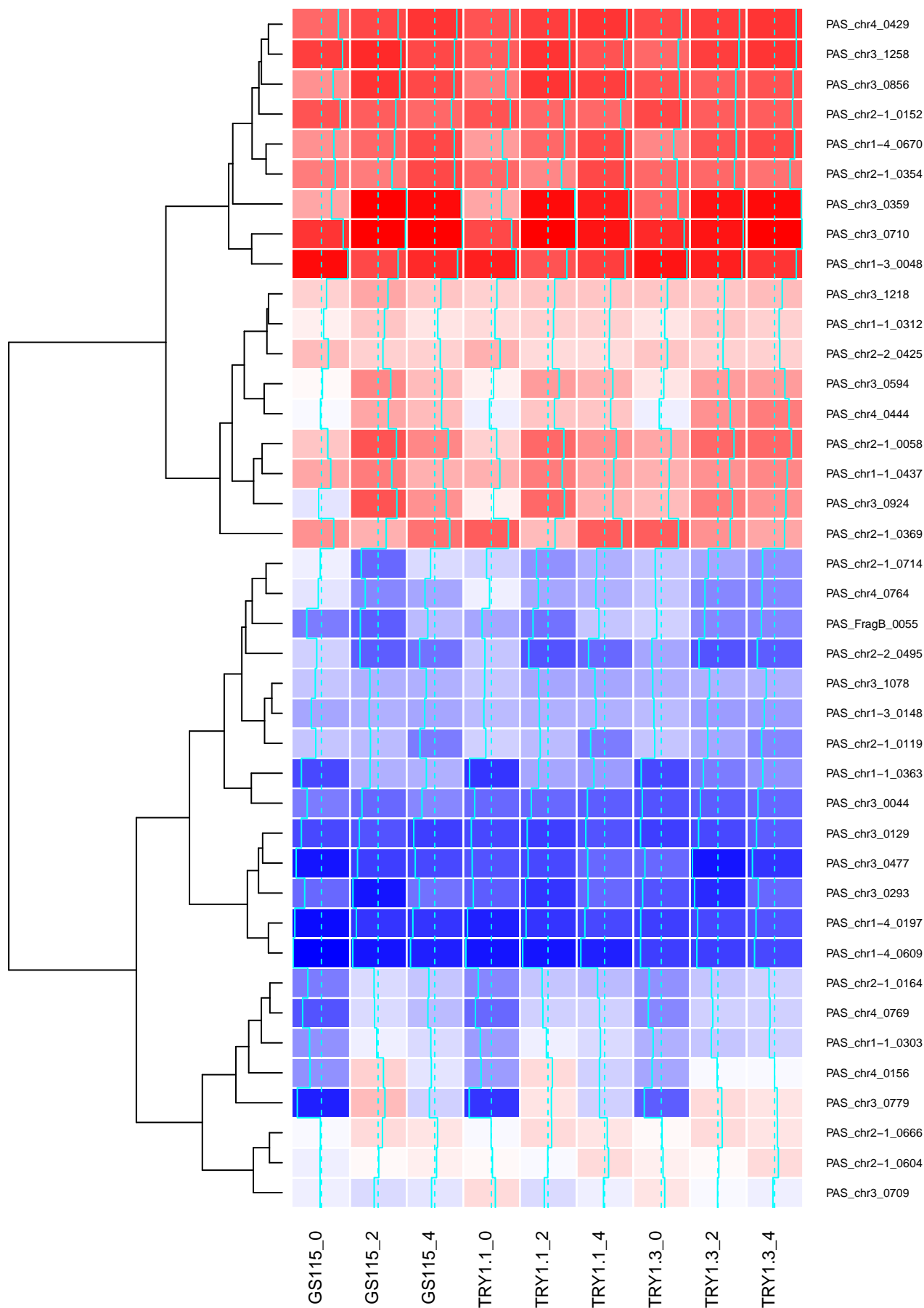

Supplement: S9 Fig — Heat maps of log 2 normalised expression levels of genes associated with ubiquitin mediated proteolysis, grouped according to KEGG pathways. Genes are uniquely identified by PAS (PAStoris) codes and expression was determined at 0, 2 and 4h after methanol addition in fed-batch cultures of GS115, TRY1-1 and TRY1-3.The associated trees cluster genes with similar expression profiles across all conditions. (PDF) [file pone.0119637.s009.pdf]

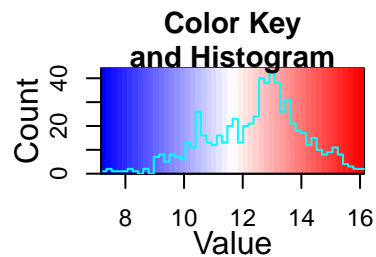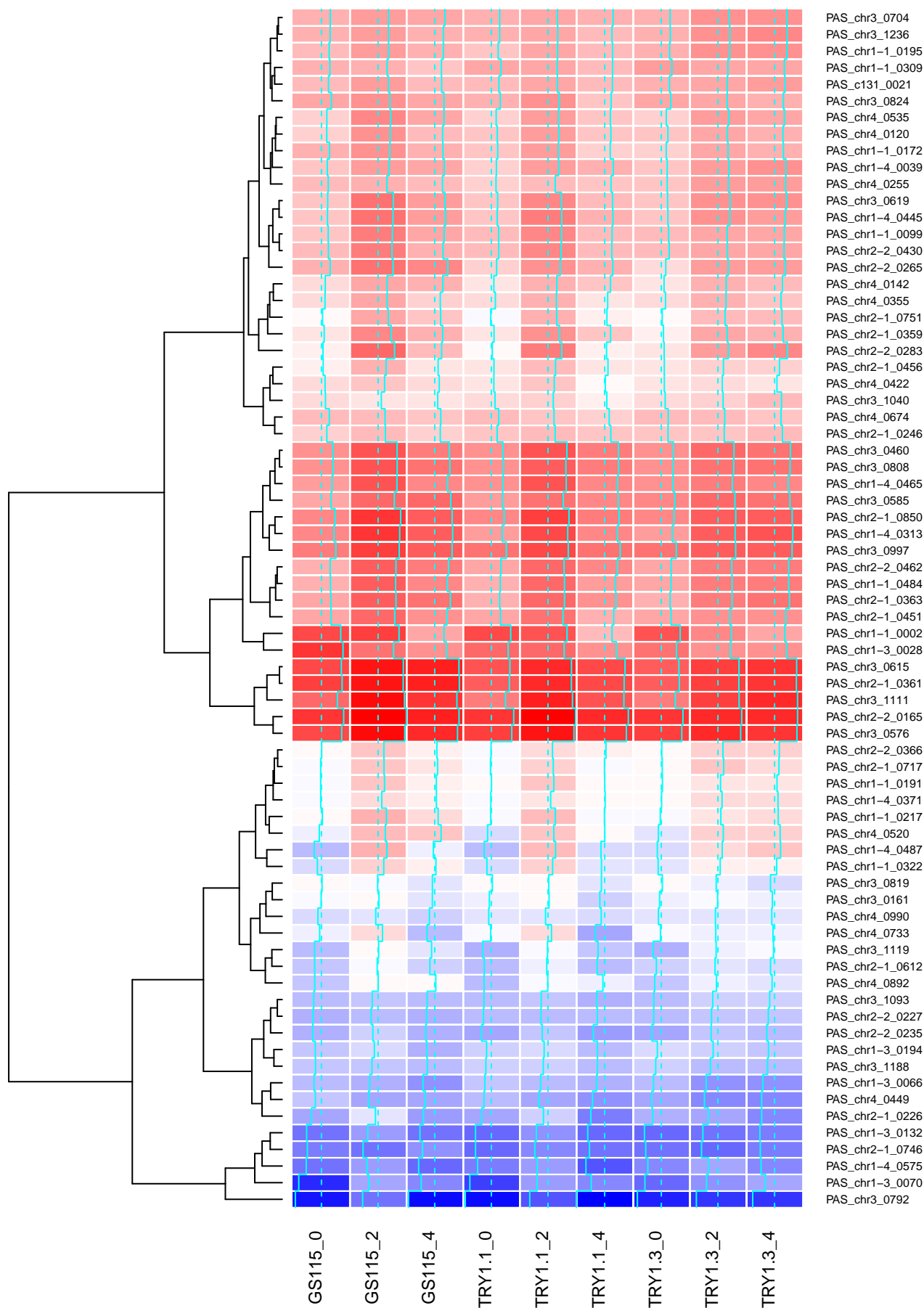

Supplement: S10 Fig — Heat maps of log 2 normalised expression levels of genes encoding proteins involved in oxidative phosphorylation, defined by KEGG families, at 2h and 4h after methanol addition in fed-batch cultures of GS115, TRY1-1 and TRY1-3, compared to those of GS115 before methanol addition. The associated trees cluster genes with similar expression profiles across all conditions. (PDF) [file pone.0119637.s010.pdf]

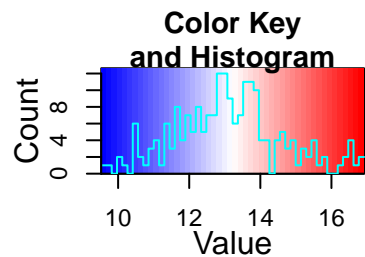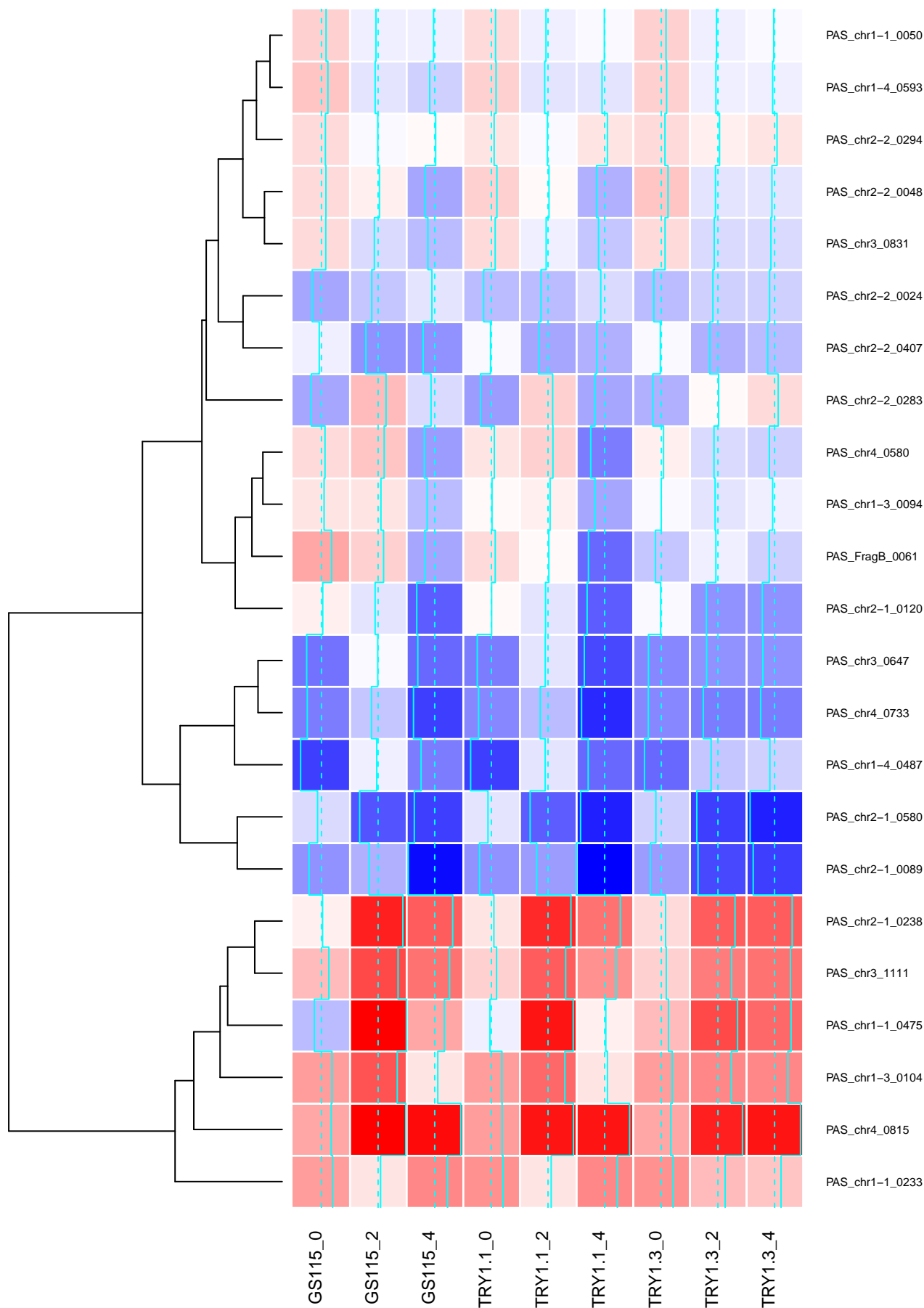

Supplement: S11 Fig — Heat maps of log 2 normalised expression levels of genes encoding proteins involved in the TCA Cycle, defined by KEGG families, at 2h and 4h after methanol addition in fed-batch cultures of GS115, TRY1-1 and TRY1-3, compared to those of GS115 before methanol addition. The associated trees cluster genes with similar expression profiles across all conditions. (PDF) [file pone.0119637.s011.pdf]

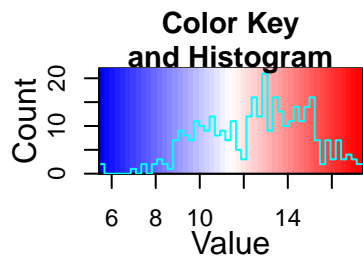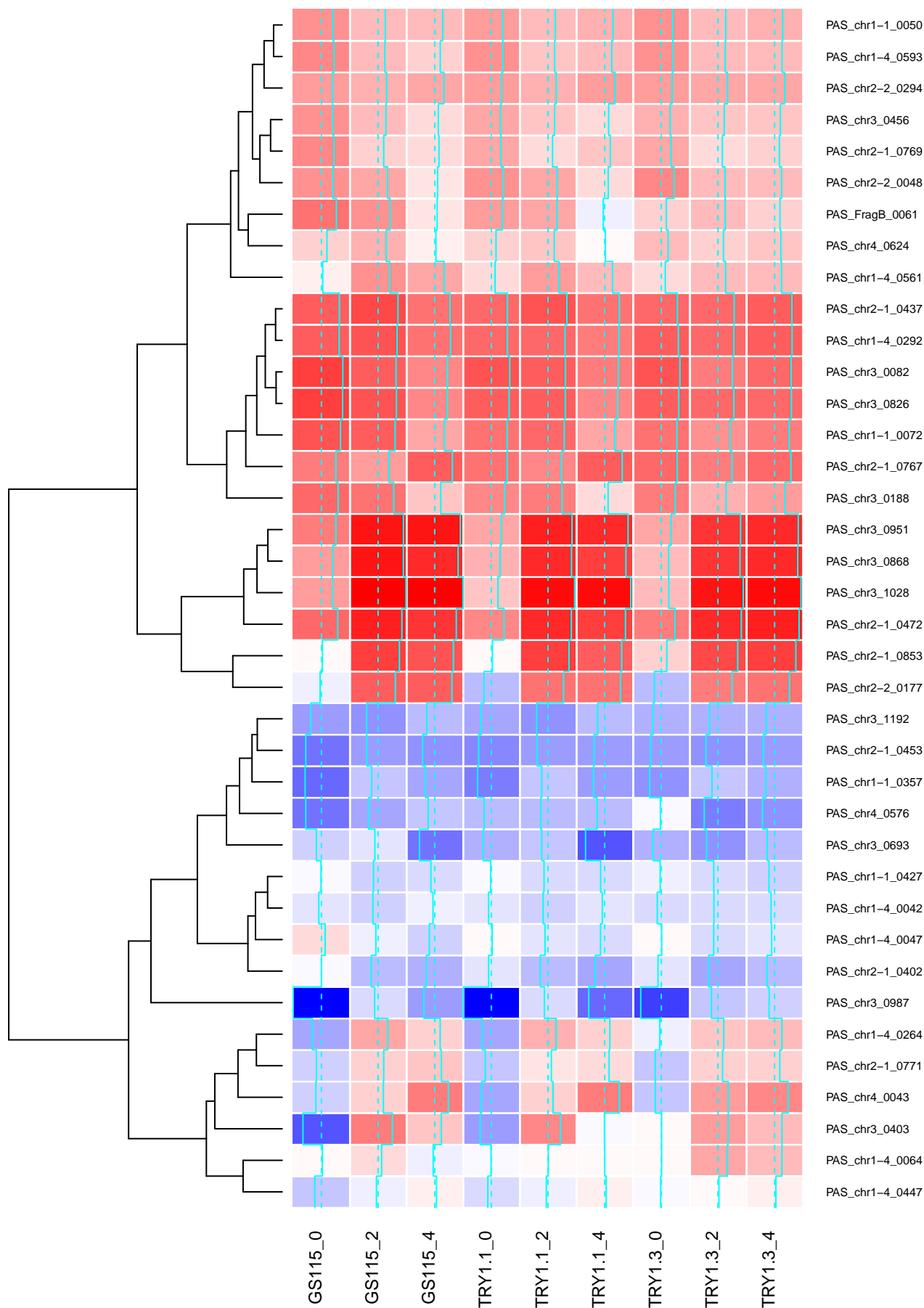

Supplement: S12 Fig — Heat maps of log 2 normalised expression levels of genes encoding proteins involved in glycolysis/gluconeogenesis, defined by KEGG families, at 2h and 4h after methanol addition in fed-batch cultures of GS115, TRY1-1 and TRY1-3, compared to those of GS115 before methanol addition. The associated trees cluster genes with similar expression profiles across all conditions. (PDF) [file pone.0119637.s012.pdf]

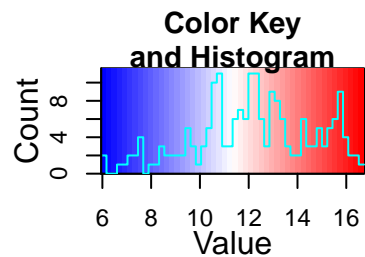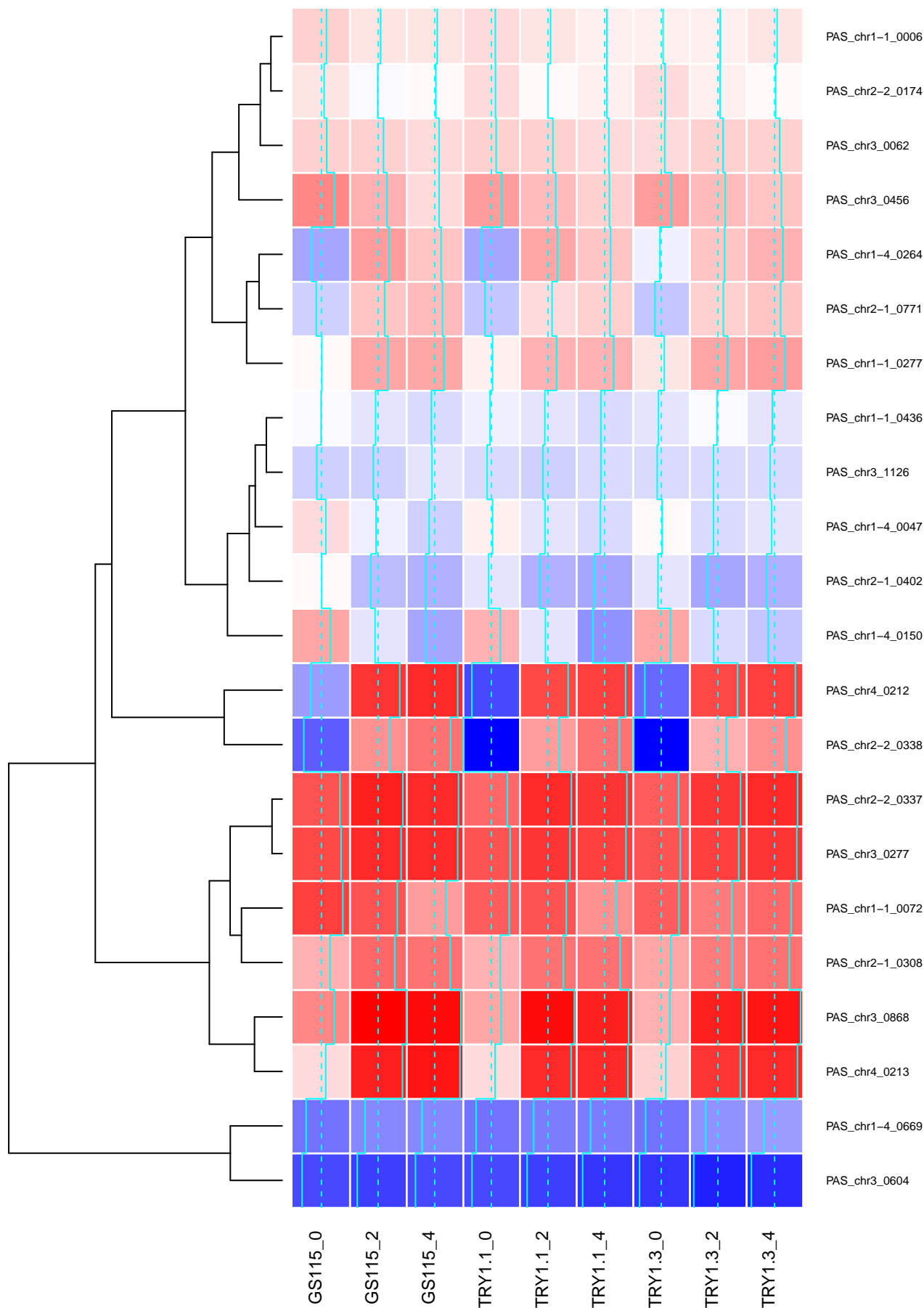

Supplement: S13 Fig — Heat maps of log 2 normalised expression levels of genes encoding proteins involved in the pentose phosphate cycle, defined by KEGG families, at 2h and 4h after methanol addition in fed-batch cultures of GS115, TRY1-1 and TRY1-3, compared to those of GS115 before methanol addition. The associated trees cluster genes with similar expression profiles across all conditions. (PDF) [file pone.0119637.s013.pdf]

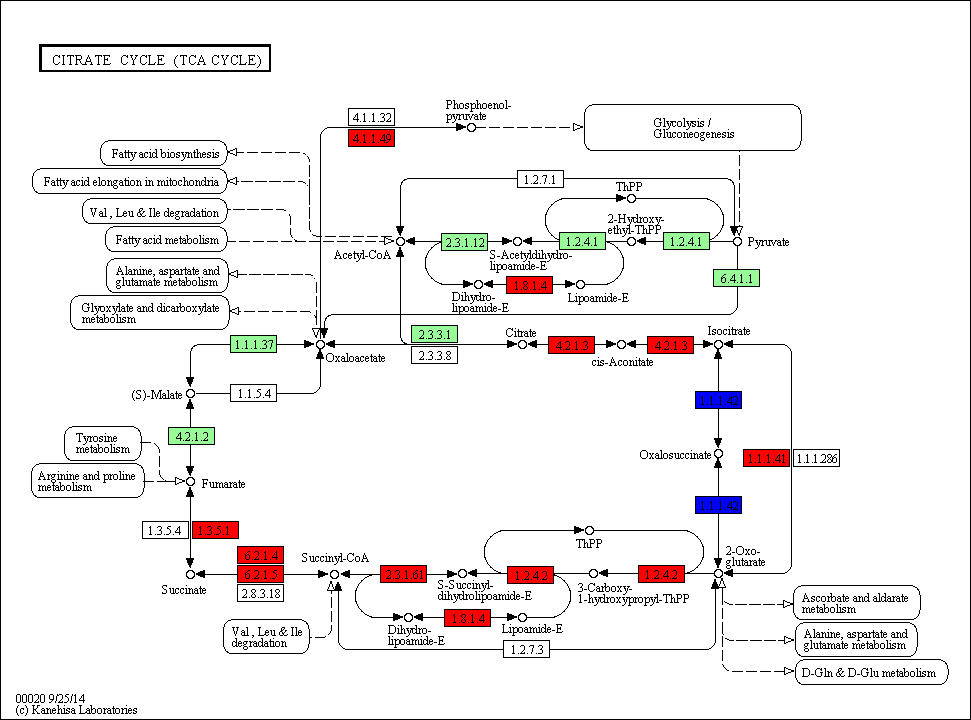

Supplement: S14 Fig — Comparisons are based on KEGG pathways. Genes where expression is significantly up-regulated (red) or down-regulated (blue) in TRY1-3 compared to GS115 are highlighted. P pastoris genes where expression is not significantly different are highlighted in green. (TIF) [file pone.0119637.s014.tif]

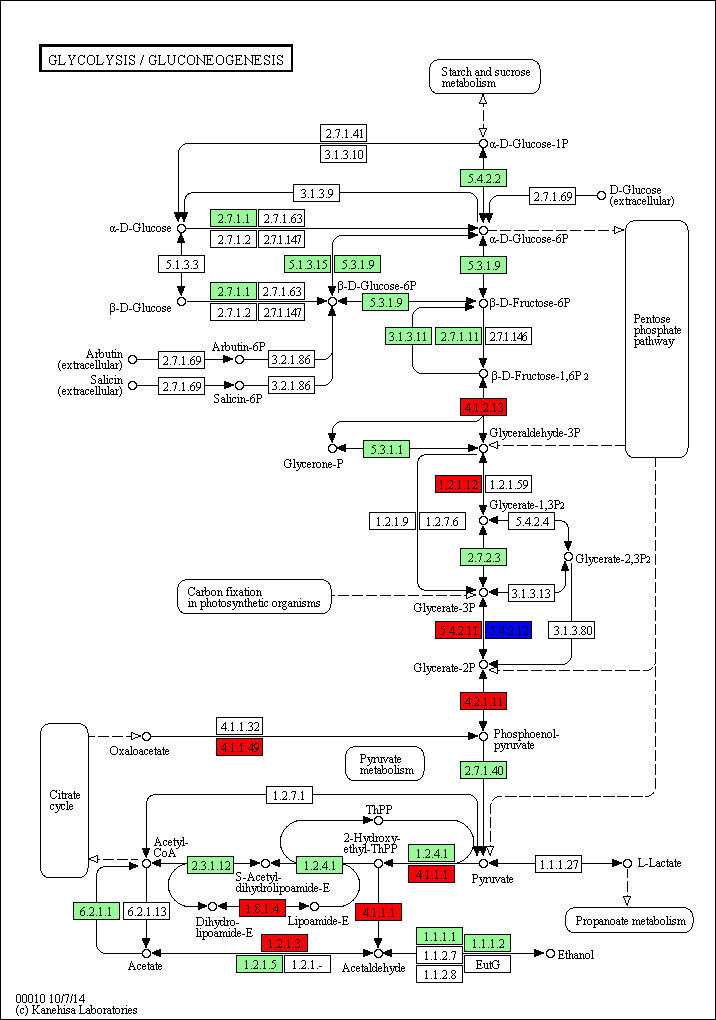

Supplement: S15 Fig — Comparisons are based on KEGG pathways. Genes where expression is significantly up-regulated (red) or down-regulated (blue) in TRY1-3 compared to GS115 are highlighted. P pastoris genes where expression is not significantly different are highlighted in green (TIF) [file pone.0119637.s015.tif]

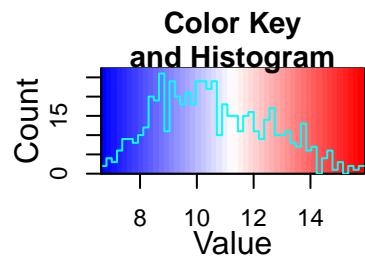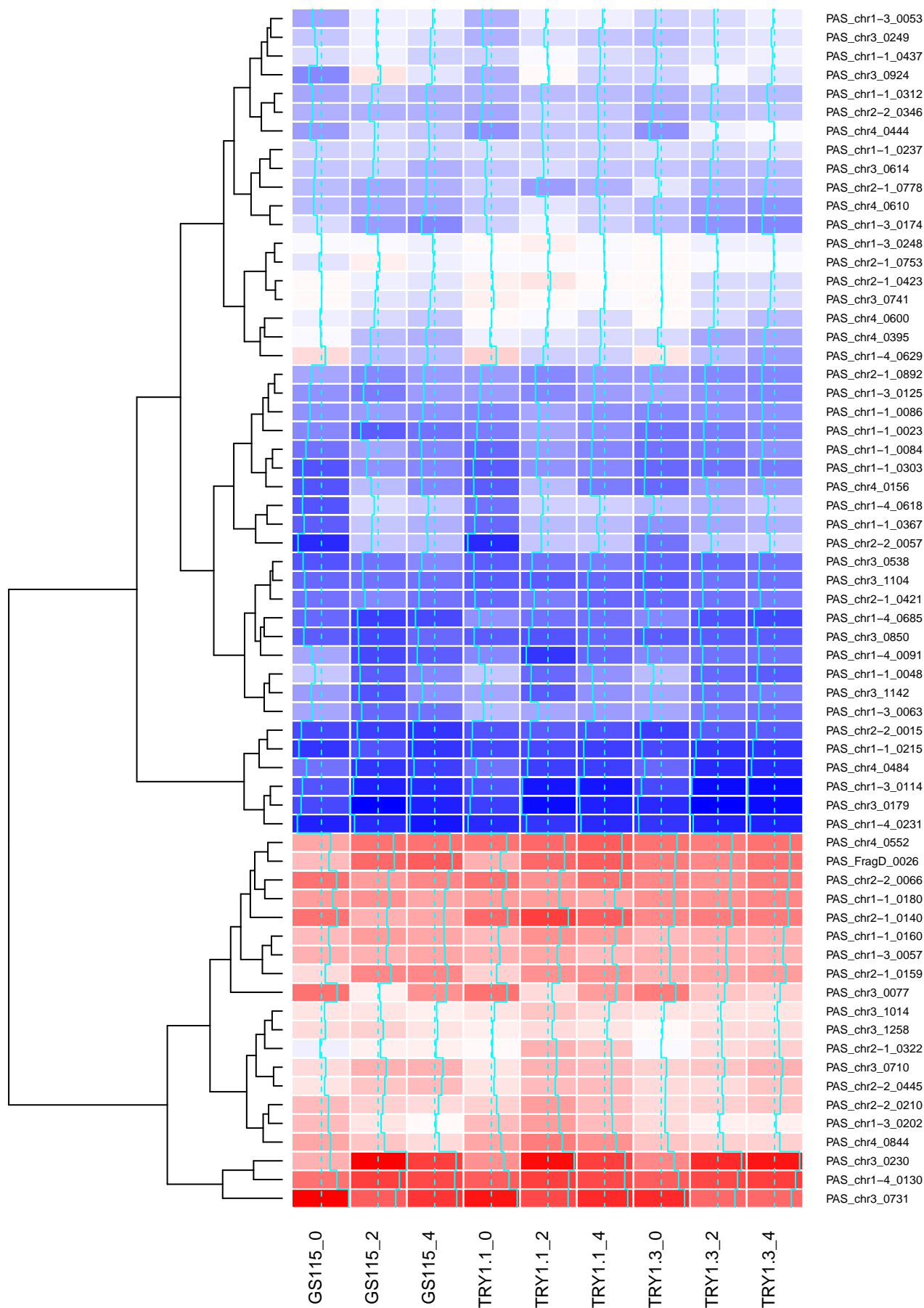

Supplement: S16 Fig — Heat maps of log 2 normalised expression levels of genes associated with protein processing in the ER, grouped according to KEGG pathways. Genes are uniquely identified by PAS (PAStoris) codes and expression was determined at 0, 2 and 4h after methanol addition in fed-batch cultures of GS115, TRY1-1 and TRY1-3. The associated trees cluster genes with similar expression profiles across all conditions. (PDF) [file pone.0119637.s016.pdf]

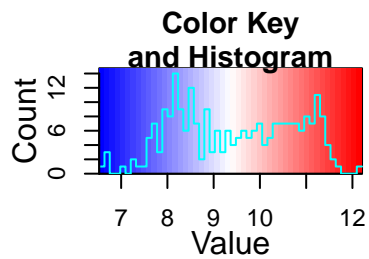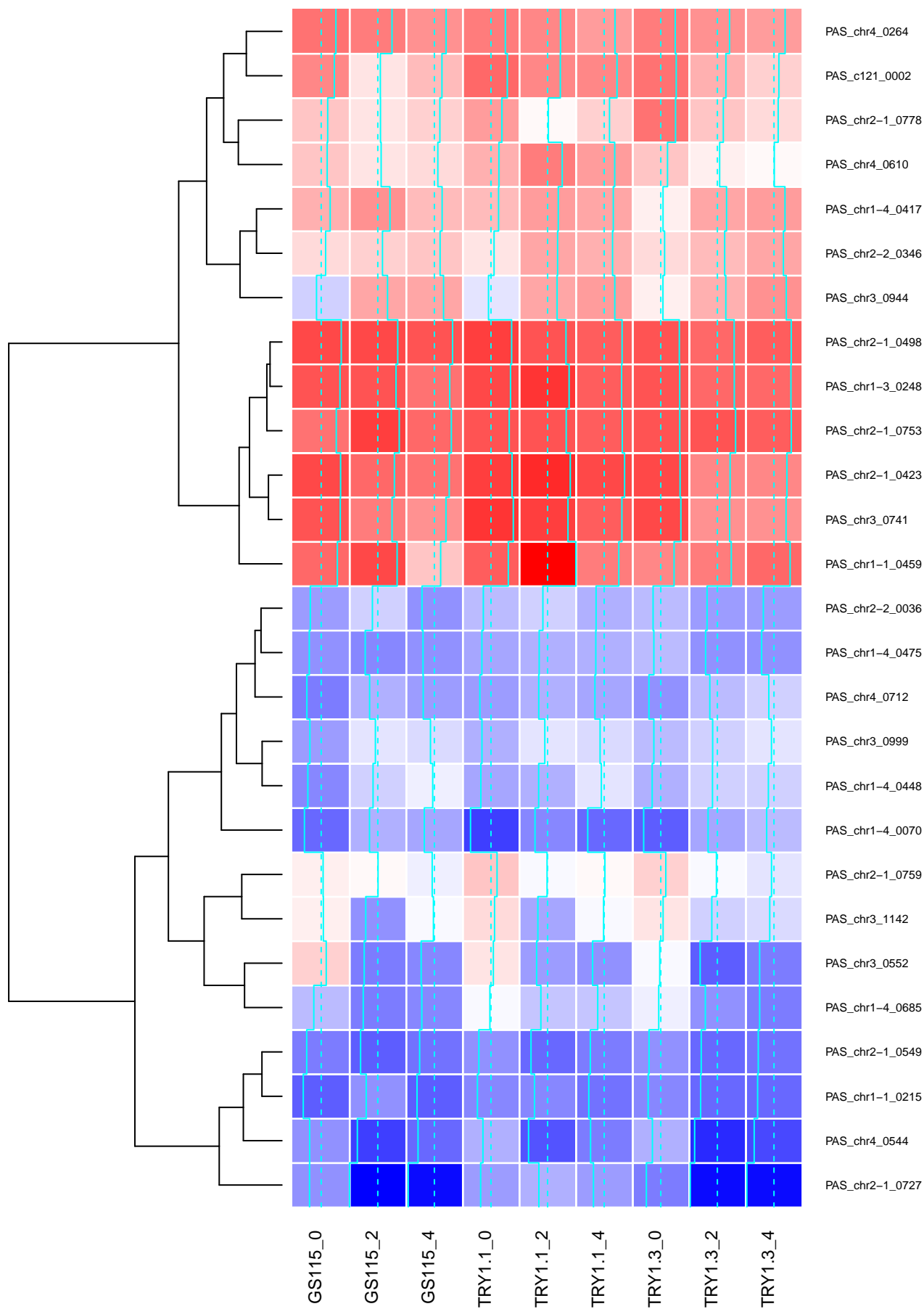

Supplement: S17 Fig — Heat maps of log 2 normalised expression levels of genes associated with N-glycan biosynthesis, grouped according to KEGG pathways. Genes are uniquely identified by PAS (PAStoris) codes and expression was determined at 0, 2 and 4h after methanol addition in fed-batch cultures of GS115, TRY1-1 and TRY1-3. The associated trees cluster genes with similar expression profiles across all conditions. (PDF) [file pone.0119637.s017.pdf]
