# Supplementary material for: Translational Arrest Due to Cytoplasmic Redox Stress Delays Adaptation to Growth on Methanol and Heterologous Protein Expression in a Typical Fed-Batch Culture of Pichia pastoris
Source: PLoS One. 2015 Mar 18;10(3):e0119637. doi: 10.1371/journal.pone.0119637 (PMC4364781; doi:10.1371/journal.pone.0119637)
Supplement: S3 Fig — Log 2 normalised expression levels of ribosomal protein biosynthesis genes grouped according to KEGG pathways. Genes are uniquely identified by PAS (PAStoris) codes and expression was determined at 0, 2 and 4h after methanol addition in fed-batch cultures of GS115, TRY1-1 and TRY1-3.The associated trees cluster genes with similar expression profiles across all conditions. (PDF) [file pone.0119637.s003.pdf]

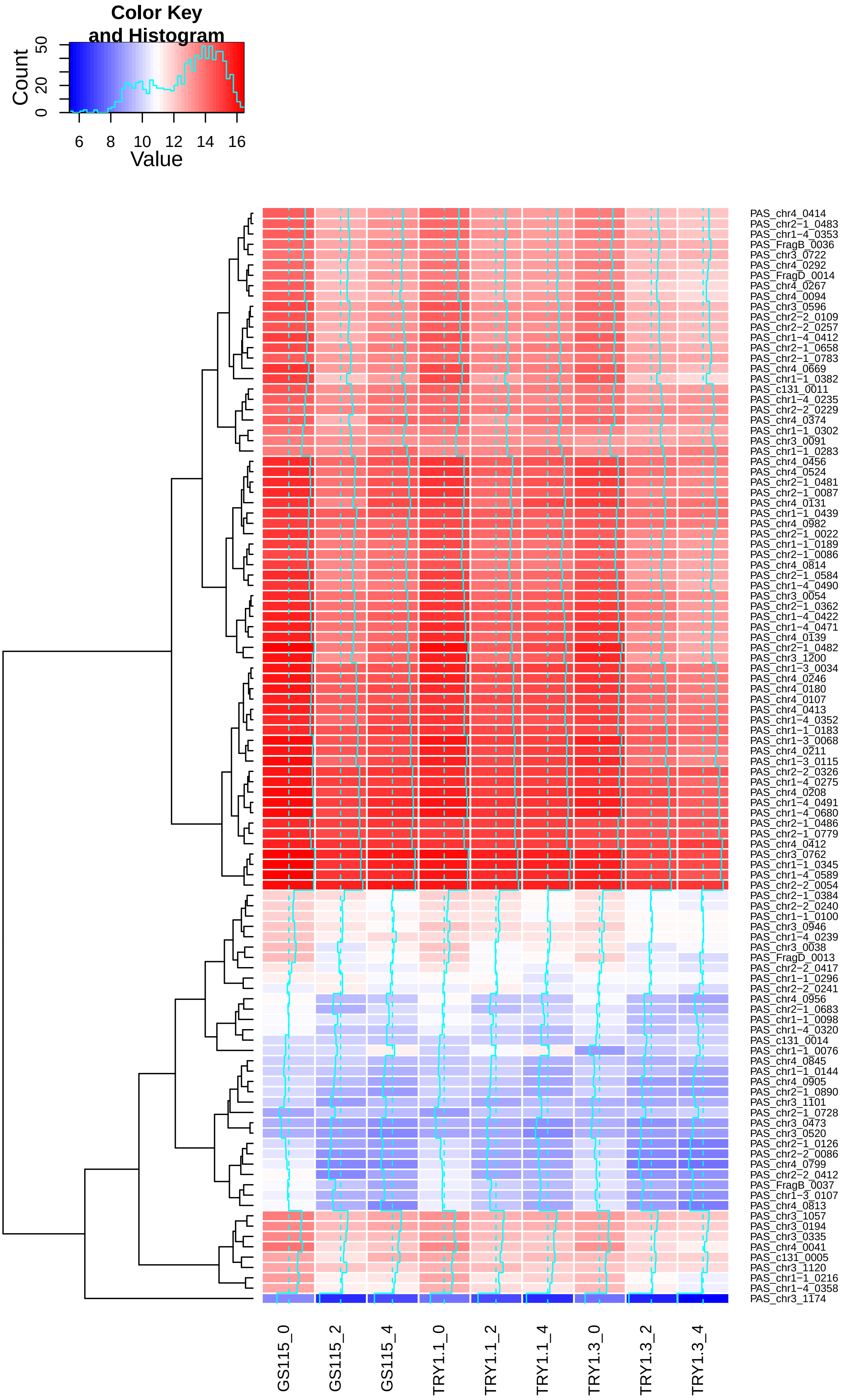
Color Key
and Histogram
Count
0
20
50
6
8
10
12
14
16
Value
PAS_chr4_0414
PAS_chr2-1_0483
PAS_chr1-4_0353
PAS_FragB_0036
PAS_chr3_0722
PAS_chr4_0292
PAS_FragD_0014
PAS_chr4_0267
PAS_chr4_0094
PAS_chr3_0596
PAS_chr2-2_0109
PAS_chr2-2_0257
PAS_chr1-4_0412
PAS_chr2-1_0658
PAS_chr2-1_0783
PAS_chr4_0669
PAS_chr1-1_0382
PAS_c131_0011
PAS_chr1-4_0235
PAS_chr2-2_0229
PAS_chr4_0374
PAS_chr1-1_0302
PAS_chr3_0091
PAS_chr1-1_0283
PAS_chr4_0456
PAS_chr4_0524
PAS_chr2-1_0481
PAS_chr2-1_0087
PAS_chr4_0131
PAS_chr1-1_0439
PAS_chr4_0982
PAS_chr2-1_0022
PAS_chr1-1_0189
PAS_chr2-1_0086
PAS_chr4_0814
PAS_chr2-1_0584
PAS_chr1-4_0490
PAS_chr3_0054
PAS_chr2-1_0362
PAS_chr1-4_0422
PAS_chr1-4_0471
PAS_chr4_0139
PAS_chr2-1_0482
PAS_chr3_1200
PAS_chr1-3_0034
PAS_chr4_0246
PAS_chr4_0180
PAS_chr4_0107
PAS_chr4_0413
PAS_chr1-4_0352
PAS_chr1-1_0183
PAS_chr1-3_0068
PAS_chr4_0211
PAS_chr1-3_0115
PAS_chr2-2_0326
PAS_chr1-4_0275
PAS_chr4_0208
PAS_chr1-4_0491
PAS_chr1-4_0680
PAS_chr2-1_0486
PAS_chr2-1_0779
PAS_chr4_0412
PAS_chr3_0762
PAS_chr1-1_0345
PAS_chr1-4_0589
PAS_chr2-2_0054
PAS_chr2-1_0384
PAS_chr2-2_0240
PAS_chr1-1_0100
PAS_chr3_0946
PAS_chr1-4_0239
PAS_chr3_0038
PAS_FragD_0013
PAS_chr2-2_0417
PAS_chr1-1_0296
PAS_chr2-2_0241
PAS_chr4_0956
PAS_chr2-1_0683
PAS_chr1-1_0098
PAS_chr1-4_0320
PAS_c131_0014
PAS_chr1-1_0076
PAS_chr4_0845
PAS_chr1-1_0144
PAS_chr4_0905
PAS_chr2-1_0890
PAS_chr3_1101
PAS_chr2-1_0728
PAS_chr3_0473
PAS_chr3_0520
PAS_chr2-1_0126
PAS_chr2-2_0086
PAS_chr4_0799
PAS_chr2-2_0412
PAS_FragB_0037
PAS_chr1-3_0107
PAS_chr4_0813
PAS_chr3_1057
PAS_chr3_0194
PAS_chr3_0335
PAS_chr4_0041
PAS_c131_0005
PAS_chr3_1120
PAS_chr1-1_0216
PAS_chr1-4_0358
PAS_chr3_1174
GS115_0
GS115_2
GS115_4
TRY1.1_0
TRY1.1_2
TRY1.1_4
TRY1.3_0
TRY1.3_2
TRY1.3_4
