# Supplementary material for: Translational Arrest Due to Cytoplasmic Redox Stress Delays Adaptation to Growth on Methanol and Heterologous Protein Expression in a Typical Fed-Batch Culture of Pichia pastoris
Source: PLoS One. 2015 Mar 18;10(3):e0119637. doi: 10.1371/journal.pone.0119637 (PMC4364781; doi:10.1371/journal.pone.0119637)
Supplement: S5 Fig — Heat maps of log 2 normalised expression levels of tRNA synthase genes. Genes are uniquely identified by PAS (PAStoris) codes and expression was determined at 0, 2 and 4h after methanol addition in fed-batch cultures of GS115, TRY1-1 and TRY1-3.The associated trees cluster genes with similar expression profiles across all conditions. (PDF) [file pone.0119637.s005.pdf]

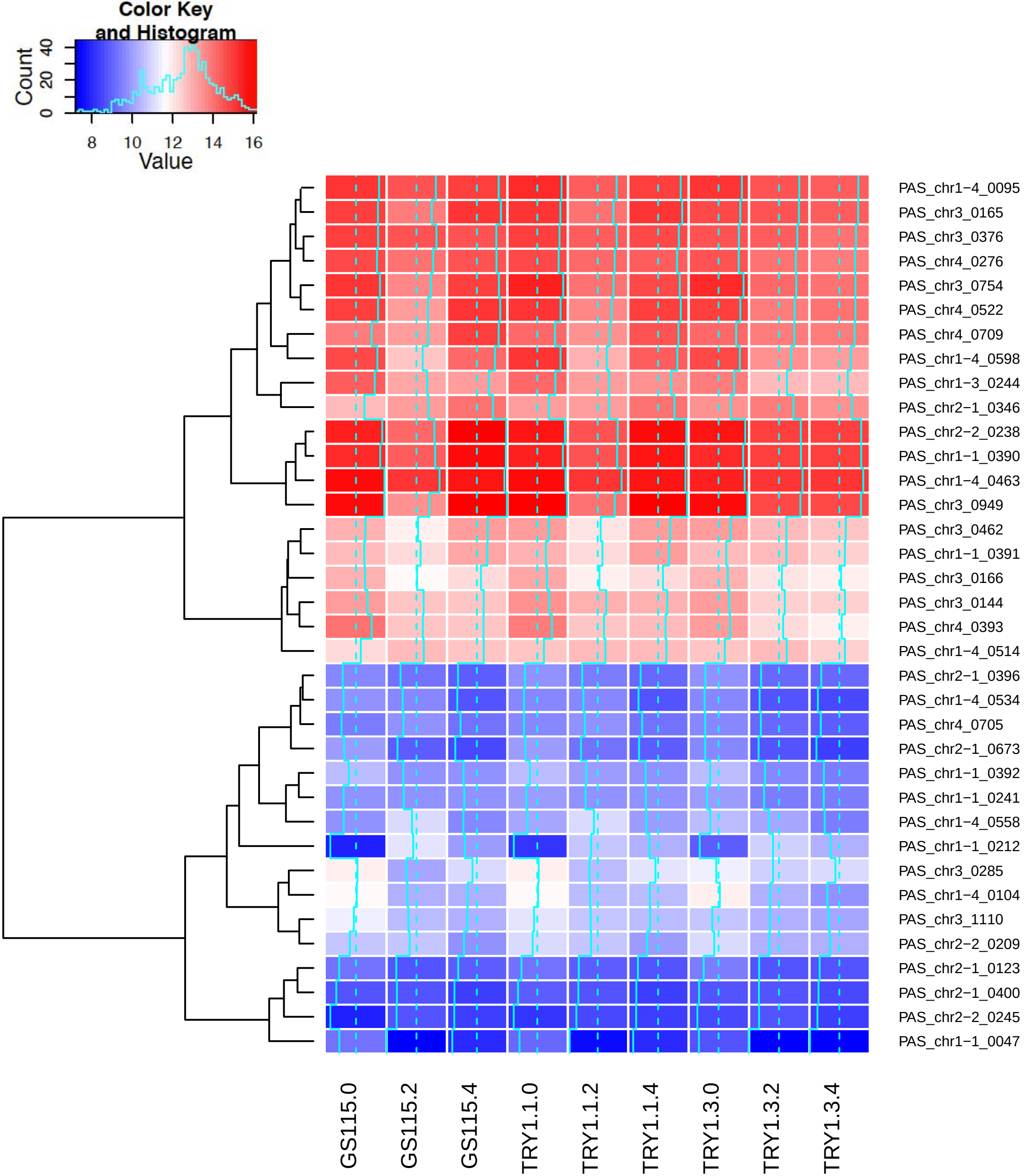
Color Key
and Histogram
Count
0
20
40
8
10
12
14
16
Value
PAS_chr1−4_0095
PAS_chr3_0165
PAS_chr3_0376
PAS_chr4_0276
PAS_chr3_0754
PAS_chr4_0522
PAS_chr4_0709
PAS_chr1−4_0598
PAS_chr1−3_0244
PAS_chr2−1_0346
PAS_chr2−2_0238
PAS_chr1−1_0390
PAS_chr1−4_0463
PAS_chr3_0949
PAS_chr3_0462
PAS_chr1−1_0391
PAS_chr3_0166
PAS_chr3_0144
PAS_chr4_0393
PAS_chr1−4_0514
PAS_chr2−1_0396
PAS_chr1−4_0534
PAS_chr4_0705
PAS_chr2−1_0673
PAS_chr1−1_0392
PAS_chr1−1_0241
PAS_chr1−4_0558
PAS_chr1−1_0212
PAS_chr3_0285
PAS_chr1−4_0104
PAS_chr3_1110
PAS_chr2−2_0209
PAS_chr2−1_0123
PAS_chr2−1_0400
PAS_chr2−2_0245
PAS_chr1−1_0047
GS115.0
GS115.2
GS115.4
TRY1.1.0
TRY1.1.2
TRY1.1.4
TRY1.3.0
TRY1.3.2
TRY1.3.4
