# Supplementary material for: Translational Arrest Due to Cytoplasmic Redox Stress Delays Adaptation to Growth on Methanol and Heterologous Protein Expression in a Typical Fed-Batch Culture of Pichia pastoris
Source: PLoS One. 2015 Mar 18;10(3):e0119637. doi: 10.1371/journal.pone.0119637 (PMC4364781; doi:10.1371/journal.pone.0119637)
Supplement: S7 Fig — Heat maps of log 2 normalised expression levels of genes associated with autophagy, grouped according to KEGG pathways. Genes are uniquely identified by PAS (PAStoris) codes and expression was determined at 0, 2 and 4h after methanol addition in fed-batch cultures of GS115, TRY1-1 and TRY1-3.The associated trees cluster genes with similar expression profiles across all conditions. (PDF) [file pone.0119637.s007.pdf]

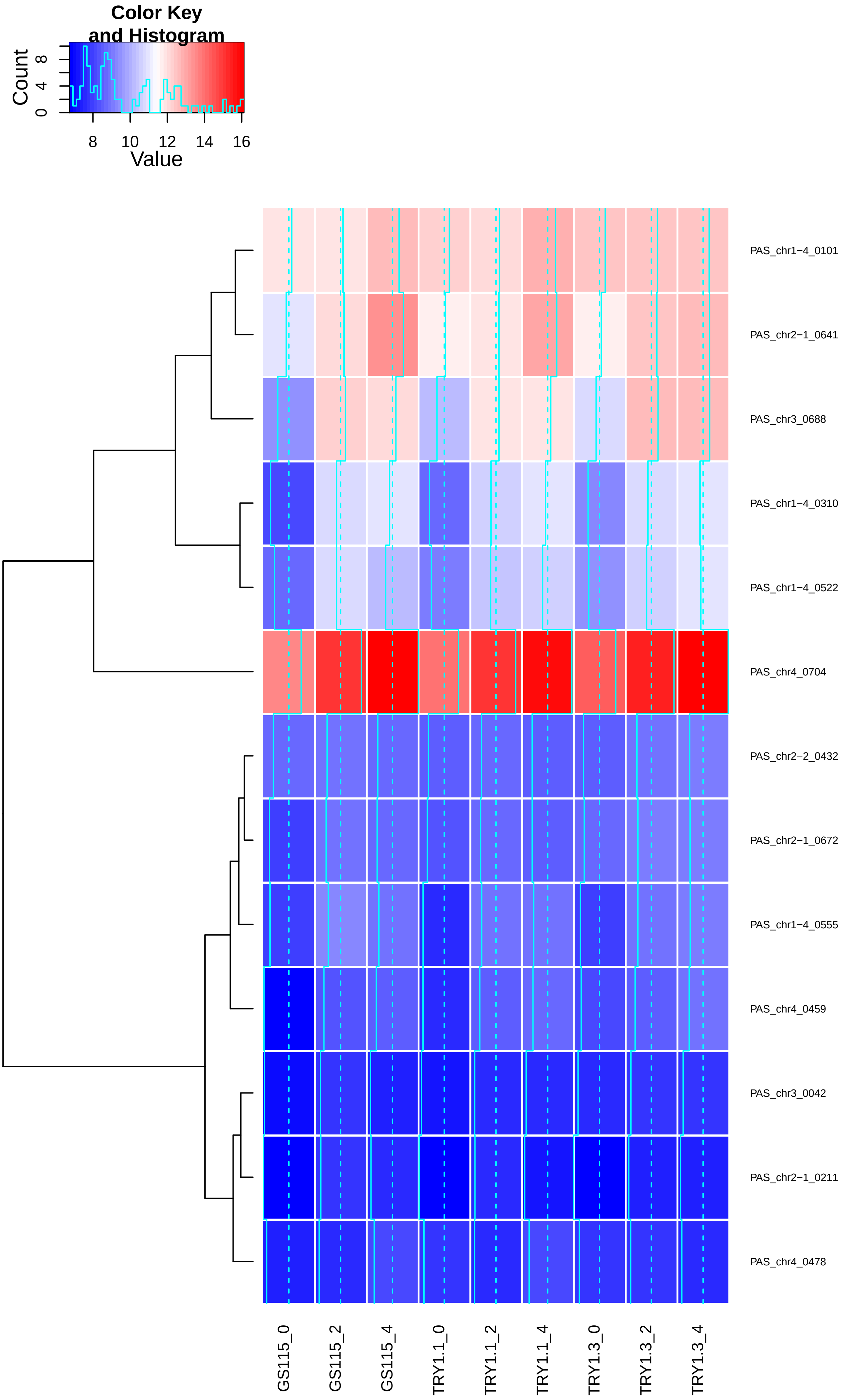

Color Key
and Histogram
Count
0
4
8
8
10
12
14
16
Value
PAS_chr1−4_0101
PAS_chr2−1_0641
PAS_chr3_0688
PAS_chr1−4_0310
PAS_chr1−4_0522
PAS_chr4_0704
PAS_chr2−2_0432
PAS_chr2−1_0672
PAS_chr1−4_0555
PAS_chr4_0459
PAS_chr3_0042
PAS_chr2−1_0211
PAS_chr4_0478
GS115_0
GS115_2
GS115_4
TRY1.1_0
TRY1.1_2
TRY1.1_4
TRY1.3_0
TRY1.3_2
TRY1.3_4
